# Spatiotemporal Variation of Ecological Quality in the Yinshan Mountains Detected by MODIS Remote Sensing Indicators

**DOI:** 10.1002/ece3.72846

**Published:** 2026-01-14

**Authors:** Zhikun Zhao, Zhigang Fang, Yunlong Zhang, Chao Ma

**Affiliations:** ^1^ School of Surveying, Mapping and Land Information Engineering Henan Polytechnic University Jiaozuo China; ^2^ School of Environment and Spatial Information China University of Mining and Technology Xuzhou China; ^3^ Key Laboratory of Spatio‐temporal Information and Ecological Remediation of Mines, Ministry of Natural Resources, Henan Polytechnic University Jiaozuo China; ^4^ Research Center for Cultivated Land Protection and High Quality Urban and Rural Development in the Yellow River Basin, Henan Polytechnic University Jiaozuo China

**Keywords:** arid and semiarid regions, desertified steppe ecosystem, MODIS RSEI, optimal parameter geographic detector (OPGD), Yinshan Mountains

## Abstract

Arid and semiarid regions constitute the primary distribution areas of desert ecosystems, and the long‐term, multifactor dynamic assessments of ecological quality can provide a scientific basis for the regional construction and sustainable development of desertified steppe ecosystems. To address the ecological vulnerability and monitoring needs of the Yinshan Mountains, we constructed a new MODIS‐based Remote Sensing Ecological Indicator (MODIS RSEI) based on MODIS data from 2001 to 2023. This indicator integrates greenness (SAVI, Soil Adjusted Vegetation Index), humidity (SWCI, Surface Water Capacity Index), dryness (NDBBI, Normalized Difference Bareness and Built‐up Index), heat (LST, Land Surface Temperature), as well as a salinity index (CSI, Comprehensive Salinity Index). Additionally, an optimal parameter geographic detector (OPGD) was employed to analyze the driving factors affecting ecological quality and their interactions. The results show that (1) the MODIS RSEI in the Yinshan Mountains exhibited a spatial pattern of “low in the west and high in the east,” fluctuating temporally between poor (0.20–0.40), moderate (0.40–0.60), and good (0.60–0.80) levels; (2) analysis of the Hurst index indicated that 62.53% of the MODIS RSEI in the Yinshan Mountains exhibited sustainable stability; and (3) single‐factor detection based on the OPGD showed that the spatial differentiation of MODIS RSEI was mainly affected by NPP (*q* = 0.837), precipitation (*q* = 0.474), and grazing intensity (*q* = 0.416). The interaction of multiple factors was significant, and the interaction of any two driving factors was greater than the influence of a single driving factor on the spatial differentiation of the Yinshan Mountains. This study provides a methodological framework and empirical evidence to support ecological conservation planning in the Yinshan Mountains, with potential applications in other arid and semiarid regions.

## Graphical abstract

1. A comprehensive ecological index MRSEI based on MODIS was proposed.
2. The new indicators of greenness, humidity, and dryness are more applicable to the ecological evaluation of the desertification steppes.
3. The introduction of comprehensive salinity index can effectively reflect the issue of soil salinization.
4. FROM‐TO vector transformation can effectively express the direction of gradient change.
5. The partitioning effect of explanatory variables can be optimized using the optimal parameter geo‐detector model.

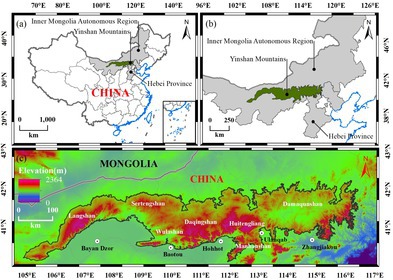

## 1 Introduction

Desertified steppe ecosystems are typical native ecosystems in arid and semiarid regions of China, where the ecological environment is the most fragile, the poverty population is the most concentrated, and it is the dust source area and the main occurrence area of sandstorms (Cheng et al. 2020). It provides important ecological services for windbreak and sand fixation, hydrological regulation, soil conservation, and biodiversity conservation (Desert Ecosystem Technical Research Project Team 2014). On September 25, 2015, the United Nations General Assembly unanimously adopted a resolution entitled “Transforming our world: the 2030 Agenda for Sustainable Development” (2030 Agenda), which included protecting, restoring, and promoting sustainable ecosystem development (SDG 15) among the 17 global Sustainable Development Goals (SDGs) (United Nations 2015). SDG 15 specifically focuses on the sustainable management of forests, restoration of degraded lands, successful fighting against desertification, reduction of degraded natural habitats, and the end of biodiversity loss. Under the guidance of this global agenda, the Chinese government adheres to the development philosophy of “innovation, coordination, green, openness, and sharing”, and actively responds to the problem of desertification through the implementation of major ecological projects, the promotion of water‐saving agricultural technologies, and the strengthening of the construction of ecological reserves; it is committed to achieving long‐term health of desert ecosystems and the continuous supply of ecological services, contributing to global ecological security and sustainable development (Anon 2016; Cui et al. 2023; Lu et al. 2020; Zhang, Chen, et al. 2024).

The management and restoration of desert ecosystems is hampered by the need to monitor large areas and the lack of local human resources, and the vast and sparsely populated nature makes it difficult for traditional ground‐based monitoring methods to cover all aspects (Wang et al. 2022). Remote sensing technology provides a scientific and accurate means for the assessment and protection of fragile ecosystems because of its high efficiency, real‐time nature, and wide coverage (Lv et al. 2023; Sun et al. 2023; Yuan et al. 2021). Commonly used remote sensing indicators such as the Normalized Difference Vegetation Index (NDVI), Bare Soil Index (BSI), and Terrain Moisture Index (TWI) have become important tools for monitoring and assessing the health of desert ecosystems (Qiao et al. 2024; Sodnomov et al. 2023; Zhang, Ma, and Liu, et al. 2024). In view of the complexity of the ecological environment, researchers have mostly used composite factors to comprehensively evaluate the ecological quality of different regions (Liu et al. 2010; Mamun and An 2022; Zhang, Zhang, and Singh, et al. 2024). For example, based on Landsat remote sensing images, Xu (2013) used Principal Component Analysis (PCA) to combine four indicators, namely, greenness (Normalized Difference Vegetation Index, NDVI), humidity (Water Environmental Temperature, WET), dryness (Normalized Difference Bare Soil Index, NDBSI), and heat (Land Surface Temperature, LST), to construct a Remote Sensing Ecological Index (RSEI) model. RSEI assessment indicators with multiple composite factors provide the model with the advantages of objectivity in weighting, high visibility of results, and convenience in obtaining indicators, providing a new way of thinking for comprehensive monitoring and assessment of regional environmental quality (Liu et al. 2023; Wang, Jiang, et al. 2024; Wang, Liu, et al. 2024).

Arid and semiarid regions account for more than 30% of the global land area and nearly 40% of the world's population. Global climate change and human activities have induced land desertification in arid and semiarid regions (Huang et al. 2019; Wang et al. 2021). Low vegetation coverage and soil salinization lead to a decrease in soil and water permeability, which can easily lead to the aggravation of desertification (Gorokhova and Pankova 2024). In recent years, scholars have proposed several improved models based on the remote sensing ecological index (RSEI) to adapt to the ecological characteristics of arid and semiarid regions. For example, Zhao, Li, and Sun (2023) added the salinity index (NDSI, normalized difference salinity index) based on the RSEI and coupled it with greenness, humidity, dryness, and heat index to establish an AWRSEI model suitable for the Daihai Lake Basin. It is undeniable that the model considers the general soil salinization problem in arid and semiarid regions but ignores the influence of soil background when extracting NDVI in low vegetation coverage areas, which limits the accuracy of the model. Luo et al. (2023) used the SAVI instead of NDVI as the greenness index of the improved remote sensing ecological index DRSEI to evaluate the ecological quality of Gulang County, Gansu Province, so as to reduce the interference of soil background on vegetation index extraction. However, the model still uses the WET index in the original RSEI to reflect the comprehensive humidity of the surface and does not fully consider the low vegetation coverage in arid and semiarid regions. The high soil reflectivity may lead to the WET index not being sensitive enough to reflect humidity, and it is difficult to accurately distinguish the small humidity changes. At the same time, Diwu et al. (2022) constructed a SA‐RSEI suitable for Yongdeng County, Gansu Province on the basis of RSEI, but ignored the interference of water pixels on the PCA process. The extreme spectral characteristics of water will distort the weight distribution of PCA, resulting in the generated index more reflecting the binary contrast of “water‐land” rather than the internal quality gradient of terrestrial ecosystems. This may not only overestimate the ecological quality around the waters but also weaken the sensitivity of the index to subtle changes in key land elements such as vegetation and soil. In addition, the mRSEI model for the Qaidam Basin has distorted the results of ecological quality assessment due to the misunderstanding of the PCA principle (Jia et al. 2021). Although these improved studies have improved the applicability of RSEI in the arid region of Northwest China to a certain extent, there are still three limitations: (1) lack of regional universality—most models are designed for specific small areas and have poor mobility in the vast and heterogeneous arid regions of Northwest China; (2) the systematic monitoring of salinization stress is still weak, and there is a lack of a stable and widely suitable salinity index integration scheme; (3) insufficient data continuity guarantee—relying on Landsat and other data sources is easy to cause data loss due to cloud interference, which affects the integrity of long‐term series analysis.

The Yinshan Mountains are a typical arid and semiarid desertified steppe region in northern China, which is an ecologically fragile zone that is sensitive to global climate change and has an important impact on regional ecological security (Zhao et al. 2017). Scholars in the field have conducted extensive research on the ecological evolution of the Yinshan Mountains. For example, Ngam et al. (2022) analyzed the potential of cultivated land resources in the agro‐pastoral ecotone at the northern foot of the Yinshan Mountains based on a logistic regression model. Luo (2023) studied the community types of desertified steppes in the Yinshan Mountains and revealed the distribution characteristics of plant communities in both horizontal and vertical spaces. Liu et al. (2022) used the APSIM model to evaluate the adaptability of major crops in different precipitation years in the four major ecological regions of Inner Mongolia, including the Yinshan Mountains. Saina (2021) constructed the driving force model of annual maximum NDVI of forest, grassland, and cropland and analyzed the dynamic change trend of vegetation coverage in the north of the Yinshan Mountains. However, these studies focused on a single attribute or ecosystem index, and based on static data or short‐term observations, it is difficult to reflect the long‐term impact of dynamic factors such as climate change and human activities on the ecosystem. At the same time, the coverage of the study area is limited, and it is difficult to fully reflect the ecological quality of the Yinshan Mountains. In contrast, using a composite factor evaluation system to process long‐term series and large‐scale remote sensing data can more comprehensively evaluate ecological environmental changes in the Yinshan Mountains.

In view of this, in order to meet the needs of ecological quality assessment in arid and semiarid regions, this study proposes the following core assumptions: Can the comprehensive evaluation model constructed by integrating the optimized multisource remote sensing indicators and using long‐term sequence MODIS data reveal the spatial and temporal differentiation of ecological quality in the region and its driving mechanism more accurately and stably? To this end, this study aims to: (1) optimize the ecological indicators suitable for the environment of arid regions: greenness (SAVI), humidity (SWCI), dryness (NDBBI), heat (LST) and comprehensive salinity index (CSI); (2) construct a new remote sensing ecological index MODIS RSEI; (3) taking the Yinshan mountains as a case to verify the monitoring ability of the index to the dynamic of ecological quality from 2001 to 2023; and (4) quantify the relative contribution of natural and human factors to MODIS RSEI and reveal the driving mechanism of MODIS RSEI changes. Through the above research, this study aims to provide a scientific basis and data support for ecological protection and restoration in arid and semiarid regions.

## 2 Materials and Methods

### 2.1 Study Area Overview

**FIGURE 1:**
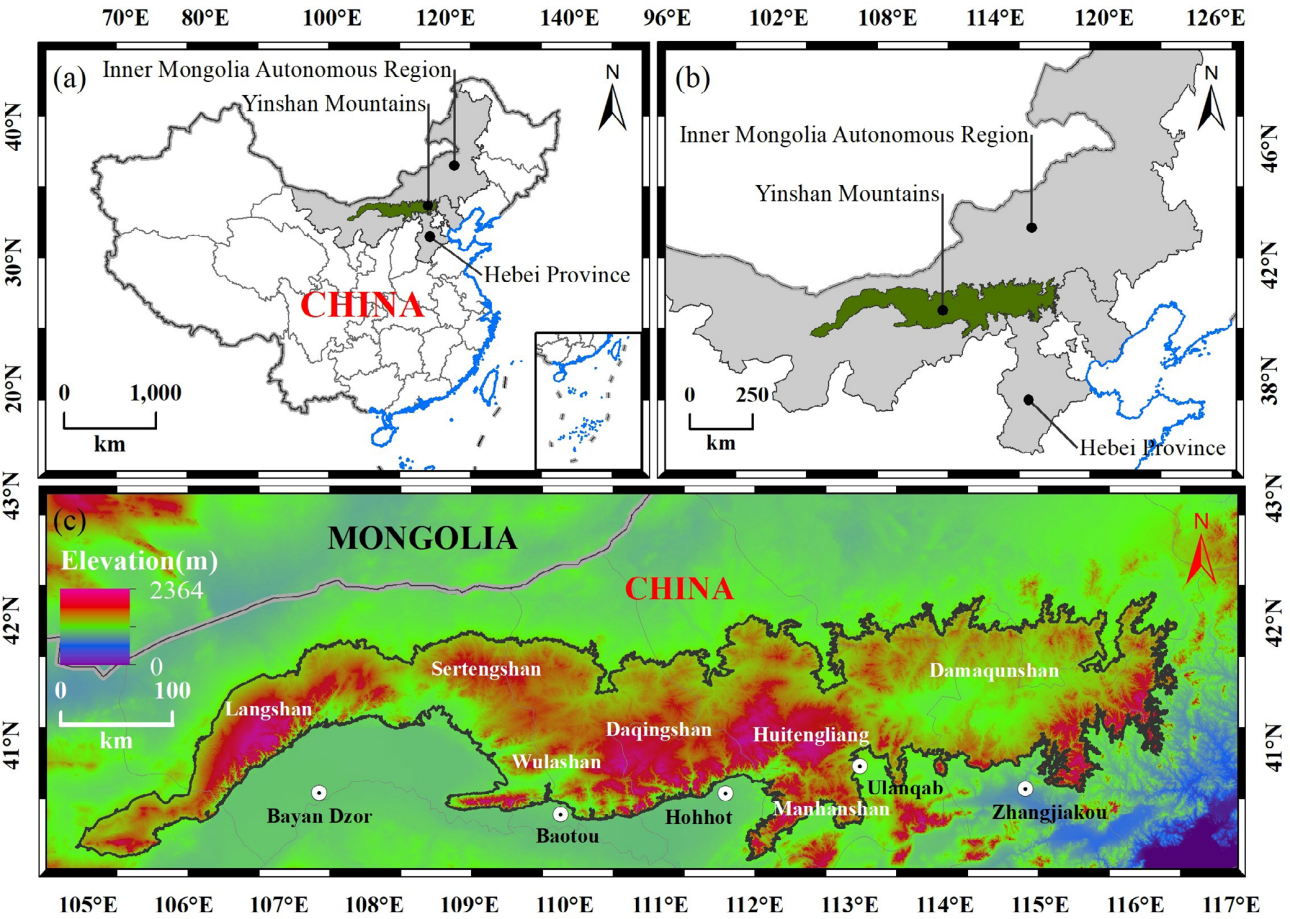
Geographic overview map of the Yinshan Mountains.

The Yinshan Mountains are mountainous areas formed by the collision of the Siberian plate and the Mongolian fold belt in the Late Jurassic period, which is blocked by the hard Ordos block in the south (Du 2009). It belongs to the east‐west tectonic system, and its terrain is gently inclined from south to north (Figure 1). The southern slope is steep, and the northern slope is gentle, between 40°–43° N and 104°–117° E. It comprises Langshan, Sertengshan, Wulashan, Daqingshan, Huitengliang, Manhanshan, and Damaqunshan. It spans the central part of the Inner Mongolia Autonomous Region and the northernmost part of Hebei Province, and the western end of the low mountain submerges into the Alxa Plateau. The eastern end is located in the upper reaches of the Luanhe River Valley to the west of Duolun (Fan 2010). It is more than 1200 km long, with an average altitude of 1500–2000 m, a mountaintop elevation of 2000–2400 m, and a cross‐sectional area of approximately 101,393 km^2^. This is the boundary between temperate monsoon and non‐monsoon regions in China. It is the watershed between the Pacific outflow water system and the Central Asian inflow water system, the natural boundary between traditional agriculture and the nomadic industry, and the boundary between the Inner Mongolia Plateau and the Loess Plateau. There is a 200 mm isohyet in the region, which has extremely important geographical significance (Chen 2016; Li 2022).

### 2.2 Data and Preprocessing

The MODIS series products from 2001 to 2023 were selected for this study, including:

(1) The surface reflectance data provided by MOD09A1 v6.1 (500 m resolution, 8‐day synthesis) were used to calculate greenness, dryness, and other indicators;
(2) The surface temperature data provided by MOD11A2 v6.1 (1 km resolution, 8‐day synthesis) were used as the heat indicator;
(3) The land use/cover change (LUCC) data provided by MCD12Q1 v6.1 (500 m resolution, annual census) were used to assist analysis and verification;
(4) The net primary productivity (NPP) data of vegetation provided by MOD17A3HGF v6.1 (500 m resolution, annual census) were used to assist analysis;
(5) Evapotranspiration (ET) data provided by MOD16A3GF v6.1 (500 m resolution, annual census) were used to assist analysis.

The study area covers two MODIS tiles (i.e., h25v04 and h26v04). To ensure the comparability of the research results, MODIS images used were selected from similar time frames (July 1 to August 31). During data selection, we prioritized dates with low cloud cover within the study area (ideally < 5%) and ensured that the selected images corresponded to the peak vegetation growth period to meet the quality requirements for subsequent analyses. The selected datasets were provided by the United States Geological Survey (USGS) Earth Resources Observation System (EROS) Data Center (https://ladsweb.modaps.eosdis.nasa.gov) and corrected for atmospheric effects such as aerosols and Rayleigh scattering (Vermote 2021).

The vector data for the study area were derived from closed vector boundaries traced along the 1200 m and 1400 m contour lines of the Yinshan Mountains. The digital elevation model (DEM) utilized data from the Shuttle Radar Topographic Mission (SRTM3 DEM, http://srtm.csi.cgiar.org) with a horizontal resolution of 90 m.

Other data: Meteorological data were derived from the ERA5‐Land reanalysis dataset of the European Center for Medium‐Range Weather Forecasts (ECMWF, https://cds.climate.copernicus.eu), with a spatial resolution of 0.25° × 0.25° (i.e., 30 × 30 km), and the study mainly used temperature (Temp., 2 m temperature) and precipitation (Prep., total precipitation) data. Based on the monthly average temperature and precipitation raster data, the 12‐month mean temperature and precipitation values were calculated to obtain the annual average temperature and precipitation raster data. Slope data were obtained from the Geospatial Data Cloud (https://www.gscloud.cn). Population density (Pop.) data were obtained from the LandScan platform (https://landscan.ornl.gov). Nighttime light intensity (NLI) data used the Extended VIIRS‐Like Artificial Nighttime Night Dataset of China (1986–2024) provided by the National Tibetan Plateau Science Data Center (https://data.tpdc.ac.cn) (Tian et al. 2025). Grazing intensity (GI) data used the Long‐term High‐resolution Dataset of Grasslands Grazing Intensity in China provided on the Figshare website (https://figshare.com) (Wang, Peng, et al. 2024). Global High‐resolution (1 km × 1 km) Soil Salinity Data and Historical 1 km Resolution Ecological Environment Quality Data of China (CHEQ) from Zenodo (https://zenodo.org) were introduced to validate the reliability of the model (Wang 2025; Xu et al. 2021) (Table 1).

**TABLE 1:** Sources and descriptions of data.

No.	Name	Time scale	Time resolution	Spatial resolution	Source
1	MOD09A1 v6.1	2001–2023	8 days	500 m × 500 m	United States Geological Survey Earth Resources Observation System (https://ladsweb.modaps.eosdis.nasa.gov)
2	MOD11A2 v6.1	2001–2023	8 days	1 km × 1 km	
3	MCD12Q1 v6.1 (LUCC)	2001–2023	Yearly	500 m × 500 m	
4	MOD17A3HGF v6.1 (NPP)	2023	Yearly	500 m × 500 m	
5	MOD16A3GF v6.1 (ET)	2023	Yearly	500 m × 500 m	
6	Temp. & prep. ERA5‐land	2023	Monthly	0.25° × 0.25°	European Center for Medium‐Range Weather Forecasts (https://cds.climate.copernicus.eu)
7	SRTM3 DEM	/	/	90 m × 90 m	CGIAR‐CSI Geo‐Portal (http://srtm.csi.cgiar.org)
8	Slope	/	/	90 m × 90 m	Geospatial Data Cloud (https://www.gscloud.cn)
9	Population density (Pop.)	2023	Yearly	1 km × 1 km	American Oak Ridge National Laboratory (https://landscan.ornl.gov)
10	Nighttime light intensity (NLI)	2023	Yearly	1 km × 1 km	National Tibetan Plateau Science Data Center (https://data.tpdc.ac.cn)
11	Grazing intensity (GI)	2023	Yearly	250 m × 250 m	Digital Science Inc. (https://figshare.com)
12	Soil salinity	2023	Yearly	1 km × 1 km	European Nuclear Research Center (CERN) (https://zenodo.org)
13	CHEQ	2023	Yearly	1 km × 1 km	

### 2.3 Methods

**FIGURE 2:**
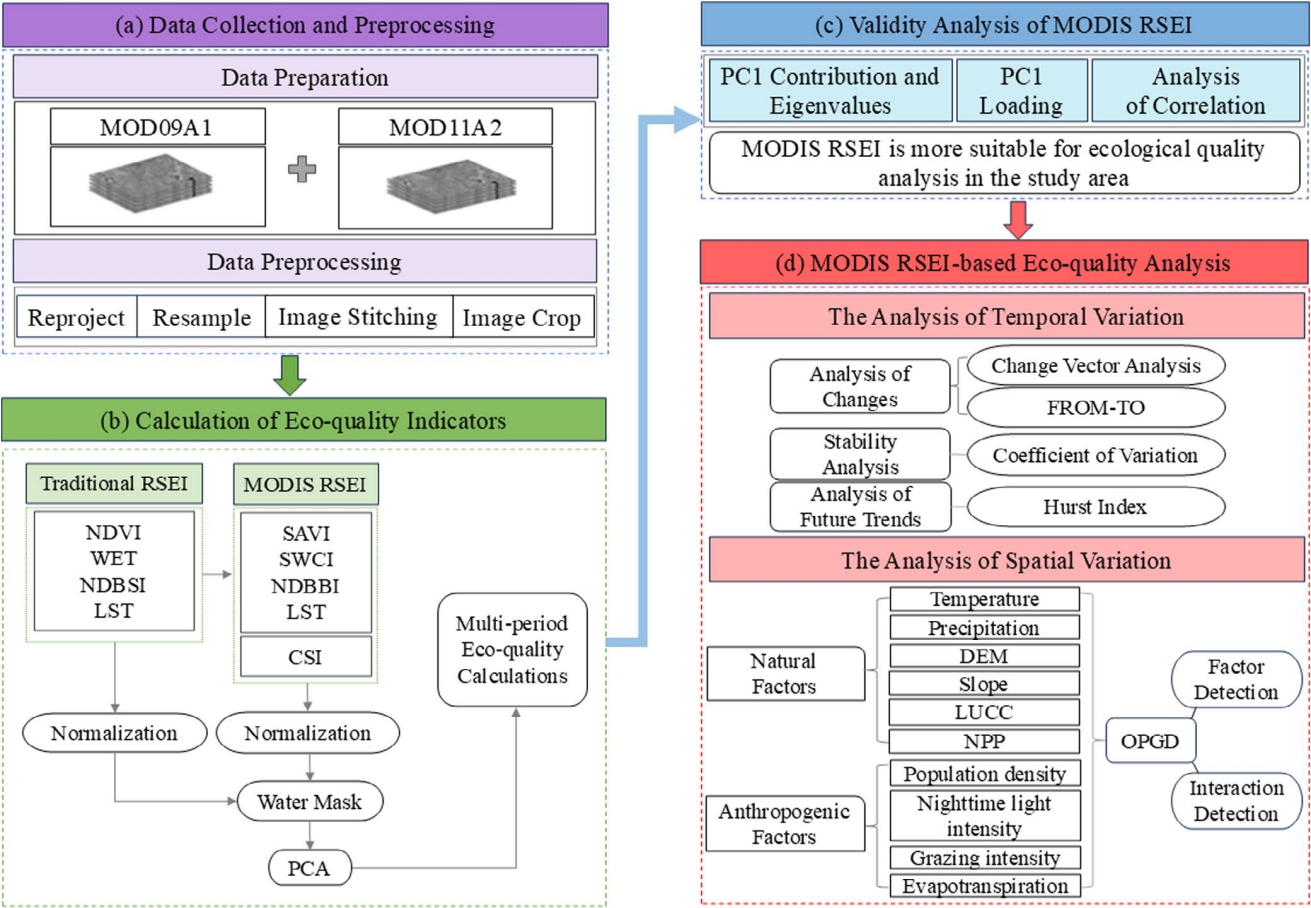
Technical flow chart.

The primary technical route used in this study is illustrated in Figure 2.

Remote Sensing Data Collection and Preprocessing: MODIS data spanning from 2001 to 2023 were downloaded and subjected to preprocessing steps, including reprojection into the UTM‐WGS84 coordinate system, resampling using the nearest neighbor interpolation method at a resolution of 500 m, image mosaicking, and clipping. These preprocessed data served as the foundation for subsequent ecological indicator calculations.

Indicator Calculation and Model Construction: Utilizing MOD09A1 v6.1 data, we computed greenness indicator (SAVI), humidity indicator (SWCI), dryness indicator (NDBBI), and comprehensive salinity indicators (CSI). MOD11A2 v6.1 data were used to derive the heat indicator (LST). A MODIS‐based Remote Sensing Ecological Indicator (MODIS RSEI) was constructed using Principal Component Analysis (PCA). Prior to model integration, an improved Modified Normalized Difference Water Index (MNDWI) was applied to mask water bodies in the study area to eliminate their impact on water bodies and normalize the indicators, ensuring uniformity in their dimensions.

Model Comparison: The MODIS RSEI was validated by comparing the first principal component (PC1) contribution rates, eigenvalues, loadings, and correlation coefficients between the traditional RSEI and MODIS RSEI. This comparative analysis aimed to demonstrate the enhanced capabilities of the MODIS RSEI.

Results Analysis: The change vector analysis (CVA), FROM‐TO vector transformation, coefficient of variation (CV), and Hurst index method were used to analyze the evolution law of ecological quality. The optimal parameter geographic detector (OPGD) model was used to explore the driving factors affecting ecological quality and their interactions.

#### 2.3.1 MODIS‐based Remote Sensing Ecological Indicator

To avoid the influence of large water bodies on the normalization and the effect of the eigenvalues of humidity and dryness on the MODIS RSEI calculations, the water bodies of the study area were masked using the Modified Normalized Difference Water Index (MNDWI) prior to the calculation of the indices, and only the ecological quality (MODIS RSEI) of the non‐water bodies was assessed (Xu 2006).

Greenness Indicator (SAVI). In arid and semiarid regions, vegetation coverage is usually low, and the bare soil background will seriously interfere with traditional vegetation indices (such as NDVI), resulting in exaggerated or distorted vegetation information. The Soil Adjusted Vegetation Index (SAVI) can effectively reduce the background noise of soil by introducing the soil adjustment coefficient *L*, and can more truly reflect the biomass and its changes of sparse vegetation. It is a better index to evaluate the vegetation coverage and growth status in arid regions (Huete 1988).

(1) $$\text{SAVI}=\frac{\left(1+L\right)\times \left(\rho_{\mathrm{nir}}-\rho_{\mathrm{red}}\right)}{\left(\rho_{\mathrm{red}}+\rho_{\mathrm{nir}}+L\right)}=\frac{\left(1+L\right)\times \left(b_{2}-b_{1}\right)}{\left(b_{1}+b_{2}+L\right)}$$

where $\rho_{\mathrm{nir}}$ is the near‐infrared band reflectance; $\rho_{\mathrm{red}}$ is the red band reflectance; $b_{1}$ and $b_{2}$ are the first and second bands of MOD09A1 v6.1, respectively. *L* is the soil adjustment parameter, and the empirical value is 0.5, which is used to reduce the influence of soil surface reflection.

Humidity Indicator (SWCI). Water is the most important limiting factor in arid ecosystems. Although the Tasseled Cap Transform (WET) is commonly used, its sensitivity to soil moisture is insufficient under low vegetation coverage conditions, and the WET derived from Lands data cannot be directly applied to MODIS sensors (Xu 2013; Zuo et al. 2022). The Surface Water Content Index (SWCI) is based on the strong absorption characteristics of liquid water by shortwave infrared spectroscopy. It is highly sensitive to soil surface water content and can effectively indicate the degree of water stress and soil drought suffered by vegetation. It is an ideal indicator for monitoring drought stress and ecological vulnerability.

(2) $$\begin{array}{c} \text{SWCI}=\frac{\rho_{\text{swir}1}-\rho_{\text{swir}2}}{\rho_{\text{swir}1}+\rho_{\text{swir}2}}=\frac{b_{6}-b_{7}}{b_{6}+b_{7}} \end{array}$$

where $\rho_{\text{swir}1}$ and $\rho_{\text{swir}2}$ are shortwave infrared band reflectance; $b_{6}$ and $b_{7}$ are the 6th and 7th bands of MOD09A1 v6.1, respectively.

Dryness Indicator (NDBBI). In arid and semiarid regions, the surface coverage types are mainly bare soil, sandy land, low coverage grassland, and sporadic towns. The traditional dryness index, Normalized Difference Bare Soil Index (NDBSI, combined with SI and IBI), is susceptible to interference in high vegetation coverage or water‐bearing areas (Xu 2013). The Normalized Difference Bareness and Built‐up Index (NDBBI) effectively suppresses the interference of vegetation and water signals by optimizing the band combination and can more accurately monitor the encroachment of desertification (natural process) and urban expansion (human process) on ecological space. It is more suitable for extracting bare soil and surface characteristics of built‐up areas in arid and semiarid regions (Zhao, Tan, et al. 2023).

(3) $$\begin{array}{c} \text{NDBBI}=\frac{1.5\rho_{\text{swir}1}-\frac{\rho_{\mathrm{nir}}+\rho_{\text{green}}}{2}}{1.5\rho_{\text{swir}1}+\frac{\rho_{\mathrm{nir}}+\rho_{\text{green}}}{2}}=\frac{1.5b_{6}-\frac{b_{2}+b_{4}}{2}}{1.5b_{6}+\frac{b_{2}+b_{4}}{2}} \end{array}$$

where $\rho_{\text{swir}1}$ is the shortwave infrared band reflectance, $\rho_{\mathrm{nir}}$ is the near‐infrared band reflectance and $\rho_{\text{green}}$ is the green band reflectance; $b_{2}$, $b_{4}$ and $b_{6}$ are the second, fourth, and sixth bands of MOD09A1 v6.1, respectively.

Heat Indicator (LST). Land Surface Temperature is an important indicator of regional thermal environment and drought stress. In arid and semiarid regions, higher surface temperature is usually closely related to vegetation water stress, evapotranspiration reduction, and rapid soil moisture loss, which is a driving factor and an important indicator of ecological degradation. In this study, 1 km resolution daytime Land Surface Temperature data (LST_Day_1km) provided by MOD11A2 product was used and converted to Celsius (Li et al. 2016).

(4) $$\begin{array}{c} \mathrm{LST}=0.02\mathrm{DN}-273.15 \end{array}$$

where the *DN* value is the gray scale value of the remote sensing image and −273.15 (°C) is absolute zero.

Comprehensive Salinity Indicator (CSI). Soil salinization is one of the most important driving factors of ecological degradation in arid and semiarid regions. The traditional RSEI framework completely ignores this key stress factor. The single salinity index has poor universality in a large area. Therefore, we innovatively constructed a Comprehensive Salinity Index (CSI), integrating three widely used salinity indices, namely, the Salinity Index‐Transient (SI‐T), the Normalized Difference Salinity Index (NDSI), and the Salinity Index 4 (SI4), to integrate multi‐index advantages and improve the robustness and reliability of large‐scale salinization monitoring (Allbed et al. 2014). When calculating, each index is normalized to the interval of [0, 1] and then the mean value is taken:

(5) $$\begin{array}{c} \mathrm{SI‐T}=\frac{\rho_{\mathrm{red}}}{\rho_{\mathrm{nir}}}\times 100=\frac{b_{1}}{b_{2}}\times 100 \end{array}$$

(6) $$\begin{array}{c} \text{NDSI}=\frac{\rho_{\mathrm{red}}-\rho_{\mathrm{nir}}}{\rho_{\mathrm{red}}+\rho_{\mathrm{nir}}}=\frac{b_{1}-b_{2}}{b_{1}+b_{2}} \end{array}$$

(7) $$\begin{array}{c} \mathrm{SI}4=\frac{\rho_{\text{swir}1}}{\rho_{\mathrm{nir}}}=\frac{b_{6}}{b_{2}} \end{array}$$

(8) $$\begin{array}{c} \mathrm{CSI}=\frac{\mathrm{SI‐T}+\text{NDSI}+\mathrm{SI}4}{3} \end{array}$$

where $\rho_{\mathrm{red}}$ is the red reflectance, $\rho_{\mathrm{nir}}$ is the near‐infrared reflectance, $\rho_{\text{swir}1}$ is the shortwave infrared reflectance; $b_{1}$, $b_{2}$ and $b_{6}$ are the first, second, and sixth bands of MOD09A1 v6.1, respectively.

The units and scales of the five component indicators are different and cannot be uniformly calculated. Therefore, each ecological factor was normalized, and the final range of values was normalized to [0, 1] before the PCA was performed.

(9) $$\begin{array}{c} {\mathrm{NI}}_{i}=\frac{I_{i}-I_{\min}}{I_{\max}-I_{\min}} \end{array}$$

where NI_*i*_ is the result of the normalization of each indicator, *I*_*i*_ is the value of each indicator at the *i*‐th pixel, and *I*_min_ and *I*_max_ are the minimum and maximum values of each indicator, respectively.

The information from SAVI, SWCI, NDBBI, LST, and CSI was then pooled into the first and second principal components using PCA. To positively correlate the value of the PC1 of PCA with ecological quality, the initial MODIS RSEI was further obtained by subtracting the PC1 from 1 (Li, Li, et al. 2024). Next, *MODIS RSEI*_0_ was normalized to facilitate comparisons across metrics, using the following formula:

(10) $$\begin{array}{c} {\text{MODIS RSEI}}_{0}=1-\mathrm{PC}1\left[f\left(\text{SAVI},\text{SWIC},\text{NDBBI},\mathrm{LST},\mathrm{CSI}\right)\right] \end{array}$$

(11) $$\begin{array}{c} \text{MODIS RSEI}=\frac{{\text{MODIS RSEI}}_{0}-{\text{MODIS RSEI}}_{0\min}}{{\text{MODIS RSEI}}_{0\max}-{\text{MODIS RSEI}}_{0\min}} \end{array}$$

where MODIS RSEI_0__max_ and MODIS RSEI_0min_ are the maximum and minimum values of MODIS RSEI_0_. The closer the MODIS RSEI value is to 1, the better the ecology. According to the ecological quality status of the study area, the MODIS RSEI was divided into five classes: MODIS RSEI < 0.20 (bad), 0.20 ≤ MODIS RSEI < 0.40 (poor), 0.40 ≤ MODIS RSEI < 0.60 (moderate), 0.60 ≤ MODIS RSEI < 0.80 (good), and MODIS RSEI ≥ 0.80 (excellent).

#### 2.3.2 The Analysis of Temporal Variation

**FIGURE 3:**
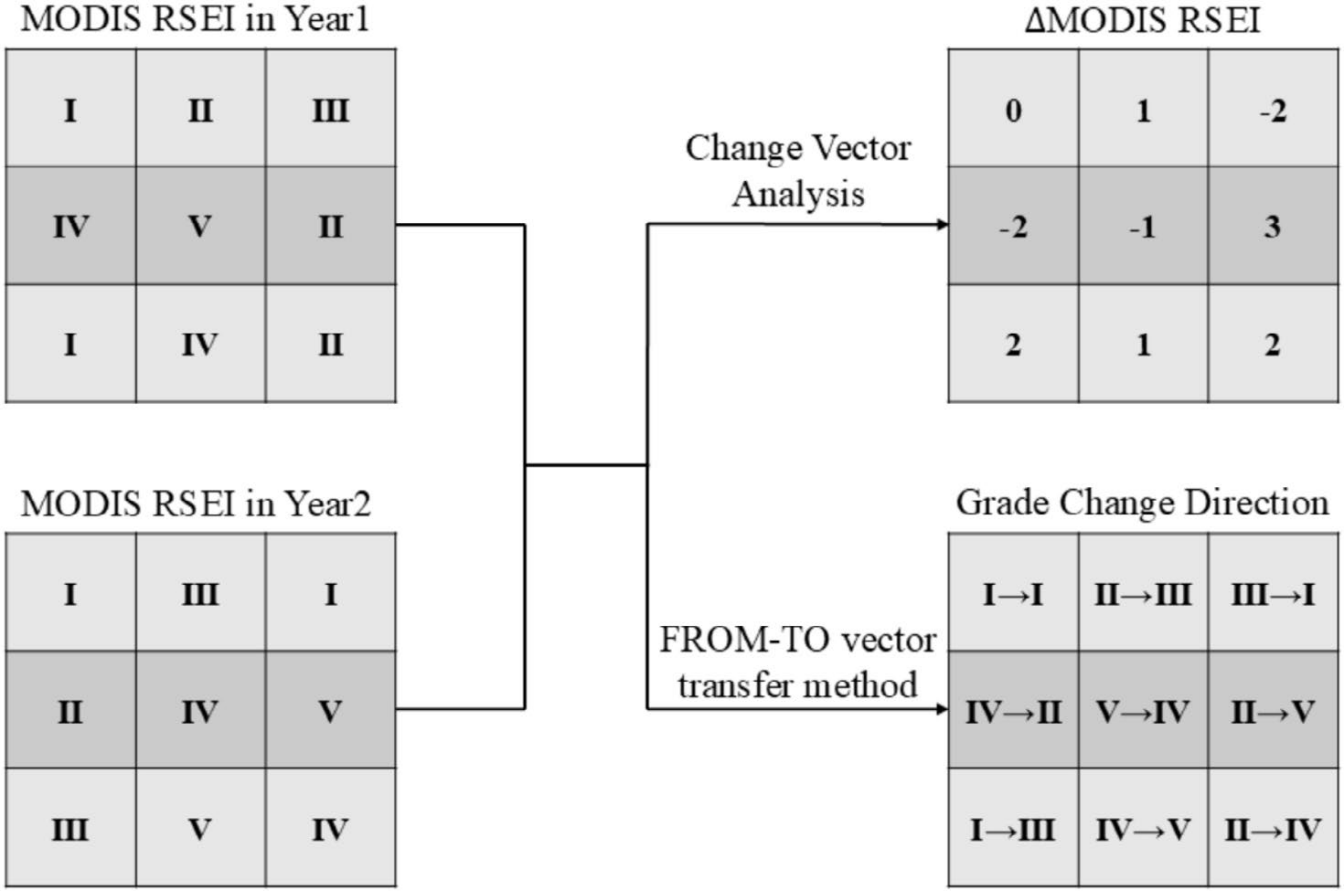
Schematic diagram of MODIS RSEI change analysis.

Change analysis of MODIS RSEI. To analyze the change degree of ecological quality in the Yinshan Mountains from 2001 to 2023, the change vector analysis (CVA) and FROM‐TO vector transfer method were used to investigate the changes in the MODIS RSEI. Taking the concept of “ecological civilization” in 2007 and the idea of “accelerating the construction of ecological civilization” in 2015 as key time nodes (Hou et al. 2021), the research period was divided into three subperiods: 2001–2007, 2007–2015, and 2015–2023. The CVA was used to identify the degree of MODIS RSEI change in each period (∆MODIS RSEI, increasing: 1, 2, 3, and 4; stable: 0; decreasing: −1, −2, −3, and −4). The direction of change in the MODIS RSEI level for each period was determined using the vector transfer method, FROM‐TO (Wu, Zhang, et al. 2024). For example, II → III denotes the MODIS RSEI level transition from II (poor) to III (moderate) (Figure 3).

Stability Analysis of MODIS RSEI. To analyze the degree of fluctuation in ecological quality in the Yinshan Mountains from 2001 to 2023, the coefficient of variation (CV) was used to evaluate the sustainable stability of the MODIS RSEI over the past 23 years. The coefficient of variation is primarily used to reflect the degree of data dispersion. The larger the value, the more discrete the data distribution and the larger the interannual fluctuation. On the contrary, the smaller the coefficient of variation, the more concentrated the data distribution, the smaller the interannual fluctuation, and the higher the time series stability (Chen et al. 2023).

(12) $$\begin{array}{c} \mathrm{CV}=\frac{\sqrt{\frac{1}{n}\sum_{i=1}^{n}\left(\text{MODIS}\;\mathrm{RSE}I_{i}-\frac{1}{n}\text{MODIS}\;\mathrm{RSE}I_{i}\right)}}{\frac{1}{n}\sum_{i=1}^{n}\text{MODIS}\;\mathrm{RSE}I_{i}} \end{array}$$

where *n* is the year, *i* is the year serial number, MODIS RSEI_*i*_ is the MODIS‐based remote sensing ecological indicator for year *i*, and CV is the coefficient of variation.

Future Trend Analysis of MODIS RSEI. To analyze the intrinsic trend characteristics of ecological quality in the Yinshan Mountains from 2001 to 2023 and predict the stability of future changes, the Hurst index (*H*) was used to explore the time series of the MODIS RSEI. The Hurst index based on the rescaled range (R/S) is an effective method for quantitatively describing the long‐term dependence of time‐series information. If 0 ≤ *H* < 0.50, the overall trend of MODIS RSEI in the future is opposite to that in the past. If *H* = 0.50, it shows that MODIS RSEI is independent of each other and has no dependence and now will not affect the future. If 0.50 < *H* ≤ 1, it indicates that the overall trend of MODIS RSEI in the future is the same as that in the past. The closer it is to 1, the stronger the persistence (Zhang and Wu 2017).

#### 2.3.3 The Analysis of Spatial Variation

Drivers analysis. The optimal parameter geographic detector (OPGD) model is a statistical model dedicated to the field of geographic information analysis that reveals the spatial heterogeneity of geographic information data and the driving forces behind it. This model includes four components: factor detection, interaction detection, ecological detection, and risk detection (Wang et al. 2010). In this study, the geographic detector model was used to measure the spatial distribution characteristics of MODIS RSEI and to detect the degree of correlation between different factors and MODIS RSEI. Its explanatory power was quantified by explaining this degree using the *q* value.

(13) $$\begin{array}{c} q=1-\frac{\sum_{n=1}^{L}N_{h}\sigma_{h}^{2}}{N\sigma^{2}} \end{array}$$

where *q* is the degree of explainability of the influence factor on the spatiotemporal variation of the RSEI, and the larger the value of *q*, the more obvious the influence of the influence factor on the MODIS RSEI. *h* is the number of classifications of the different driving factors, *L* is the number of samples of the influence factor, *N*_*h*_ and *N* are the number of units in the *h*‐th class and the entire sample, respectively, and $\sigma_{h}^{2}$ and $\sigma^{2}$ are the variances of the *h*th class and the entire sample. The larger the *q* value, the greater the influence of this factor on MODIS RSEI. However, the traditional geo‐detector adopts the discretization of subjectively determined driving factors, which has poor discretization and subjective problems (Zhao et al. 2024). Therefore, by optimizing the discretization of continuous variables in the geo‐detector, the optimal parameter geo‐detector model, OPGD, was obtained to detect the spatial heterogeneity of the Yinshan Mountains.

## 3 Results

### 3.1 Validation of MODIS RSEI

**FIGURE 4:**
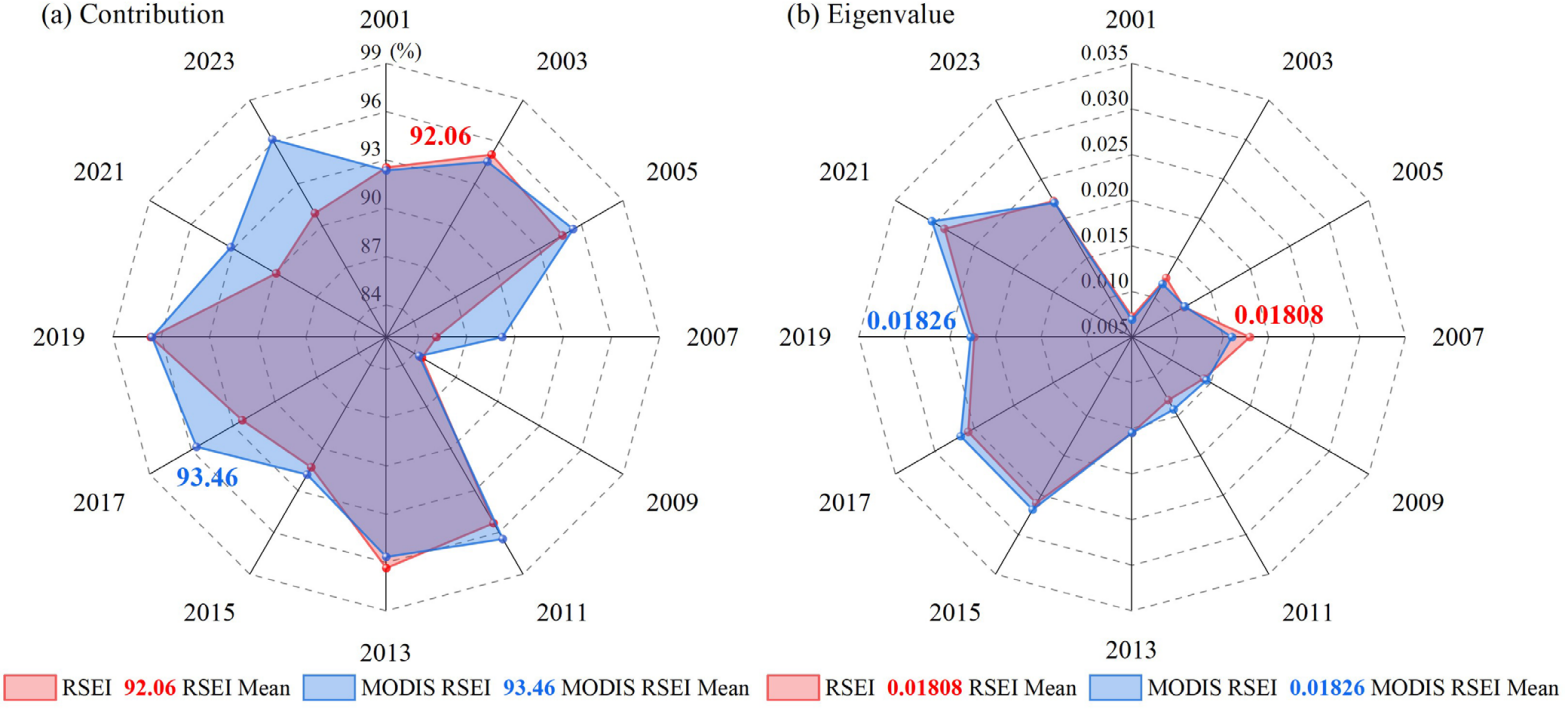
Comparison of PCA results: (a) Contribution of the two models; (b) Eigenvalues of the two models.

Traditional RSEI (based on the four ecological indicators NDVI, WET, NDBSI, and LST) and the MODIS RSEI (based on the five ecological indicators SAVI, SWCI, NDBBI, LST, and CSI) in the study area were constructed, and the PCA results of the two models were compared (Figure 4).

In all monitoring years, the contribution rate of the PC1 of MODIS RSEI was greater than 84%, and most of the characteristic information of the greenness indicator (SAVI), humidity indicator (SWCI), dryness indicator (NDBBI), heat indicator (LST), and salinity indicator (CSI) was integrated. The PC1 was used to extract the MODIS RSEI, which can effectively reflect the comprehensive status of the ecological quality in the study area. By comparing and analyzing the contribution rate of the PC1 of RSEI and MODIS RSEI in the same year, it was found that the contribution rate of the PC1 of MODIS RSEI was greater than that of RSEI in 7 of the 12 datasets from 2001 to 2023. The average contribution rate of the PC1 of MODIS RSEI was 93.46%, which was superior to the average contribution rate of the PC1 of RSEI (92.06%) (Figure 4a).

In the PCA process, the larger the eigenvalues, the more representative the original data. By comparing and analyzing the eigenvalues of RSEI and MODIS RSEI in the same year, it can be concluded that the eigenvalues of MODIS RSEI in 8 of the 12 data periods from 2001 to 2023 are greater than the eigenvalues of RSEI, and the average eigenvalues of MODIS RSEI were 1% higher than the average eigenvalues of RSEI. This shows that the MODIS RSEI eigenvalues were dominant overall (Figure 4b).

The payload of the PC1 reflects the effect of each ecological indicator on ecological quality and also reflects the rationality of the construction of the remote sensing ecological index model. In the PC1, the greenness indicator and the humidity indicator are positive; that is, they contribute positively to the ecosystem, whereas the dryness indicator and the heat indicator are negative; that is, they contribute negatively to the ecosystem. The payload values of the PC1 of MODIS RSEI are listed in Table 2, and some of the data were subjected to the “1−PC1” transformation as needed.

**TABLE 2:** Comparison of the payload values of the PC1 of RSEI and MODIS RSEI.

Year	Model	Greenness	Humidity	Dryness	Heat	Comprehensive salinity
		NDVI/SAVI	WET/SWCI	NDBSI/NDBBI	LST	CSI
2001	RSEI	−0.7217	−0.1850	0.6644	0.0584	/
	MODIS RSEI	**0.7494**	**0.2645**	−0.2088	**−0.0602**	−0.5668
2003	RSEI	−0.6691	−0.1768	0.7177	0.0774	/
	MODIS RSEI	**0.6964**	**0.2805**	−0.2748	**−0.0805**	−0.5953
2005	RSEI	−0.7180	−0.1672	0.6730	0.0607	/
	MODIS RSEI	**0.7203**	**0.2614**	−0.2198	−0.0600	−0.6008
2007	RSEI	−0.5013	−0.3920	0.4926	0.5931	/
	MODIS RSEI	**−0.5451**	−0.2863	0.1767	**0.6343**	0.4384
2009	RSEI	−0.5075	−0.1316	0.4596	0.7169	/
	MODIS RSEI	−0.5037	**−0.2194**	0.1751	0.7066	0.4101
2011	RSEI	−0.7672	−0.1485	0.6198	0.0725	/
	MODIS RSEI	0.7401	**0.2114**	−0.1974	−0.0689	−0.6031
2013	RSEI	−0.6868	−0.1588	0.7050	0.0780	/
	MODIS RSEI	**0.6900**	**0.2457**	−0.3391	**−0.0790**	−0.5851
2015	RSEI	−0.5907	−0.1180	0.5378	0.5898	/
	MODIS RSEI	−0.5869	**−0.1762**	0.2132	0.5852	0.4864
2017	RSEI	0.6884	0.4111	−0.5951	−0.0548	/
	MODIS RSEI	−0.6849	−0.3466	0.2747	0.0531	0.5767
2019	RSEI	−0.6971	−0.1329	0.7015	0.0650	/
	MODIS RSEI	−0.6969	**−0.1912**	0.3026	**0.0651**	0.6181
2021	RSEI	−0.6143	−0.3410	0.4681	0.5360	/
	MODIS RSEI	−0.6031	−0.1783	0.2347	0.5218	0.5265
2023	RSEI	0.6660	0.4331	−0.6030	−0.0675	/
	MODIS RSEI	**−0.6820**	−0.3678	0.2553	**0.0686**	0.5742

*Note:* Bold values in the MODIS RSEI rows indicate where the absolute valid factor loading exceeds that in the corresponding RSEI.

It was found that the sign of the PC1 payload was the same for both the greenness and humidity indicators in the same year, and the sign of the PC1 payload was the opposite for the dryness, heat, and comprehensive salinity indicators in the same year as their counterparts.

The correlations between each ecological indicator and RSEI and MODIS RSEI were analyzed (Table 3).

**TABLE 3:** Comparison of correlation coefficients of each ecological factor with RSEI and MODIS RSEI.

Year	Model	Greenness	Humidity	Dryness	Heat	Comprehensive salinity	Mean correlation
		NDVI/SAVI	WET/SWCI	NDBSI/NDBBI	LST	CSI	
2001	RSEI	0.9781	0.7624	−0.9721	−0.5891	/	0.8254
	MODIS RSEI	**0.9950**	**0.9552**	−0.6654	−0.5842	−0.9948	**0.8389**
2003	RSEI	0.9846	0.8473	−0.9886	−0.5569	/	0.8444
	MODIS RSEI	**0.9931**	**0.9651**	−0.8500	**−0.5615**	−0.9977	**0.8735**
2005	RSEI	0.9842	0.7637	−0.9821	−0.7270	/	0.8643
	MODIS RSEI	**0.9937**	**0.9617**	−0.7825	−0.7235	−0.9937	**0.8910**
2007	RSEI	0.9491	0.8377	−0.9425	−0.9343	/	0.9159
	MODIS RSEI	**0.9658**	**0.9509**	−0.7375	**−0.9430**	−0.9638	0.9122
2009	RSEI	0.9309	0.7470	−0.8862	−0.9368	/	0.8752
	MODIS RSEI	**0.9355**	**0.8920**	−0.6701	−0.9353	−0.9265	0.8719
2011	RSEI	0.9895	0.7880	−0.9774	−0.7199	/	0.8687
	MODIS RSEI	**0.9970**	**0.9510**	−0.7966	−0.7146	−0.9980	**0.8914**
2013	RSEI	0.9878	0.8349	−0.9892	−0.7679	/	0.8949
	MODIS RSEI	**0.9923**	**0.9615**	−0.8974	**−0.7782**	−0.9983	**0.9255**
2015	RSEI	0.9786	0.8166	−0.9550	−0.9416	/	0.9230
	MODIS RSEI	**0.9826**	**0.9442**	−0.8164	**−0.9442**	−0.9820	**0.9339**
2017	RSEI	0.9834	0.8842	−0.9776	−0.6920	/	0.8843
	MODIS RSEI	**0.9959**	**0.9581**	−0.8629	**−0.6825**	−0.9972	**0.8993**
2019	RSEI	0.9873	0.8195	−0.9882	−0.8465	/	0.9104
	MODIS RSEI	**0.9954**	**0.9500**	−0.8884	**−0.8552**	−0.9986	**0.9375**
2021	RSEI	0.9809	0.8564	−0.9603	−0.9417	/	0.9349
	MODIS RSEI	**0.9893**	**0.9590**	−0.8439	**−0.9420**	−0.9895	**0.9448**
2023	RSEI	0.9767	0.8694	−0.9797	−0.7743	/	0.9000
	MODIS RSEI	**0.9951**	**0.9723**	−0.8625	**−0.7823**	−0.9973	**0.9219**
Mean	RSEI	0.9759	0.8189	−0.9666	−0.7857	/	*p* < 0.01
	MODIS RSEI	**0.9859**	**0.9518**	−0.8601	**−0.7872**	−0.9864	

*Note:* (1) “Mean correlation” refers to the mean of the absolute values of the correlation coefficients of each ecological factor, characterizing the overall correlation of each ecological factor with the RSEI and MODIS RSEI; (2) the bold value in the MODIS RSEI row indicates that the absolute value of the correlation coefficient here exceeds the corresponding RSEI.

The results of correlation analysis (Table 3) showed that all ecological factors showed a highly significant correlation with the traditional RSEI and MODIS RSEI indexes (*p* < 0.01), and the direction of action was in line with the theoretical expectations:

(1) The greenness indicator (NDVI and SAVI) and humidity indicator (WET and SWCI) were positively correlated with RSEI and MODIS RSEI, indicating that vegetation cover and soil moisture contributed to the ecological quality of the Yinshan Mountains, while the dryness indicator (NDBSI and NDBBI) and the heat indicator (LST) were negatively correlated with RSEI and MODIS RSEI, and at the same time, the integrated salinity (CSI) was also negatively correlated with MODIS RSEI, indicating that drought, heat, and salinity negatively affect the ecological quality of the Yinshan Mountains.
(2) Compared with RSEI, the average correlation coefficients between MODIS RSEI and the greenness indicator (SAVI), humidity indicator (SWCI), and heat indicator (LST) were larger in absolute value, indicating that MODIS RSEI was more sensitive to changes in the greenness indicator, humidity indicator, and heat indicator.
(3) The introduced comprehensive salinity indicator (CSI) was negatively correlated with MODIS RSEI, with an average correlation coefficient of −0.9864, the absolute value of which was larger than that of the average correlation coefficients of the dryness indicator (NDBBI) and the heat indicator (LST). This indicates that soil salinization is one of the main factors affecting the ecological quality of the Yinshan Mountains, consistent with the findings of previous studies (Perri et al. 2022; Yagoub et al. 2023; Yang et al. 2024).
(4) The average correlation coefficients of each ecological factor with MODIS RSEI were higher than 0.82 in all years, and the average correlation coefficients of each ecological factor with MODIS RSEI were higher than those of RSEI, except for the years 2007 and 2009, indicating that MODIS RSEI was better able to characterize the comprehensive information of each ecological factor.

### 3.2 Analysis of Ecological Quality

#### 3.2.1 The Spatial Variation

**FIGURE 5:**
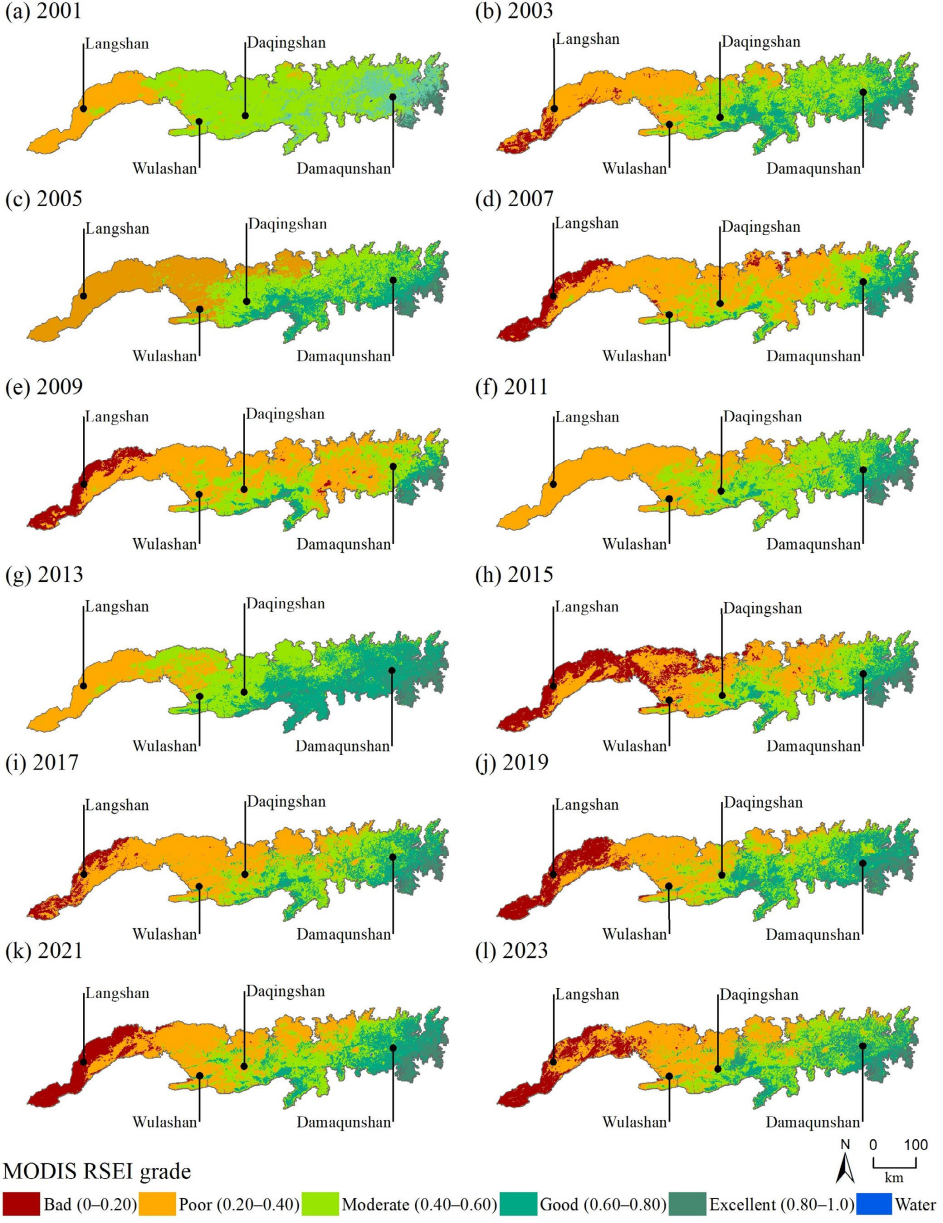
Spatial distribution of MODIS RSEI levels in the Yinshan Mountains from 2001 to 2023.

From 2001 to 2023, the MODIS RSEI in the Yinshan Mountains showed the characteristics of “lower in the west and higher in the east” ladder distribution (Figure 5). The high‐value areas were mainly concentrated in eastern Damaqunshan, southern Huitengliang, and Manhanshan. In these areas, vegetation coverage is high, precipitation is abundant, and the ecological quality is good. The low‐value areas were mainly concentrated in western Langshan and Seltengshan. In these areas, bare land is relatively concentrated, soil salinization is high, and vegetation coverage is low, resulting in poor ecological quality.

As shown in Figure 5, the low value of the MODIS RSEI shows a clear horizontal zonal distribution extending from the western Langshan region to the central and eastern Wulashan and Daqingshan regions. During 2001–2009, the ecological quality of the western region gradually deteriorated. Although it improved in 2009–2013, the improvement time was short, and ecological quality continued to decline in 2013–2023. In contrast, the ecological quality near Damaqunshan in the eastern region was relatively stable throughout the monitoring period (2001–2023), with the MODIS RSEI peaking in 2013.

The statistical results of the area percentage of each grade of MODIS RSEI (see Figure A1 for details) further reveal the evolution process of regional ecological quality. From 2001 to 2023, the proportion of regions with excellent ecological quality increased from 2.47% to 5.65%, reflecting that local ecological restoration measures have achieved initial results in some regions. The proportion of regions with good ecological quality was relatively stable, averaging approximately 20.00%. Although it fluctuated during 2007–2013, it will return to 18.86% in 2023, which is not significantly different from 16.88% in 2001. However, the proportion of regions with moderate ecological quality decreased significantly from 62.80% in 2001 to 27.00% in 2023, whereas the regions of poor and bad ecological quality increased significantly from 17.85% to 48.49% in 2023. This change is highly coincident with the two major drought events in 2005–2007 and 2018–2020 and superimposed on the vegetation destruction and soil erosion caused by the development of mineral resources in the western region, which together lead to irreversible degradation of ecological quality (Jin et al. 2022; Li et al. 2010).

Overall, the ecological quality of the Yinshan Mountains shows an overall downward trend, especially the reduction of moderate‐quality areas, and a substantial increase in poor and bad regions, indicating that the regional ecological environment is facing greater pressure.

#### 3.2.2 The Temporal Variation

To further analyze the changes in ecological quality in the Yinshan Mountains from 2001 to 2023, the study period was divided into three subperiods: 2001–2007, 2007–2015, and 2015–2023, with 2007 and 2015 as the dividing lines. Based on the CVA method, the change level in MODIS RSEI (values ranging from −4 to 4, Table 4) was calculated, and the change in MODIS RSEI was divided into five types: deteriorated significantly (−4 to −2), deteriorated (−1), unchanged (0), improved (1), and improved significantly (2 to 4).

**TABLE 4:** Regional statistics on changes in ecological conditions in the Yinshan Mountains from 2001 to 2023.

MODIS RSEI change type	MODIS RSEI change grade	2001–2007		2007–2015		2015–2023		2001–2023	
		Area (km^2^)	Percentage (%)	Area (km^2^)	Percentage (%)	Area (km^2^)	Percentage (%)	Area (km^2^)	Percentage (%)
Deteriorated significantly	−4	/	3.33	/	0.18	/	0.07	/	1.56
	−3	2.50		/		0.25		0.75	
	−2	3378.50		186.00		68.75		1584.00	
Deteriorated	−1	60316.80	59.49	18370.80	18.12	7573.75	7.43	45591.80	44.97
Unchanged	0	35102.80	34.62	64690.00	63.80	59106.80	58.29	41136.50	40.57
Improved	1	2574.25	2.54	17214.00	16.98	32184.00	31.74	11928.30	11.76
Improved significantly	2	18.00	0.02	923.75	0.92	2401.00	2.46	1151.50	1.14
	3	/		8.25		93.25		/	
	4	/		/		1.50		/	

The proportion of areas with poor and obvious ecological quality from 2001 to 2007 was as high as 62.82%, while the proportion of areas with improvement and obvious improvement was only 2.56%, indicating that the ecological degradation problem was prominent at this stage. From 2007 to 2015, the proportion of areas with poor and obvious deterioration of ecological quality decreased to 18.30%, the proportion of areas with improvement and obvious improvement increased to 17.90%, and the trend of ecological deterioration eased. During the period 2015–2023, the area of ecological quality deterioration and obvious deterioration was further reduced to 7.50%, while the area of improvement and obvious improvement was significantly increased to 34.20%, indicating that the ecological quality gradually improved from 2007 to 2023. However, from the perspective of the entire monitoring cycle (2001–2023), the total proportion of areas with obvious deterioration and deterioration of ecological quality was 46.53%, whereas the proportion of areas with improvement and obvious improvement was only 12.90%. This shows that although the ecological quality has improved after 2007, the overall ecological quality of the Yinshan Mountains has degraded in the past 23 years, and the ecological quality of some areas has decreased by at least one level.

From the perspective of spatial evolution, during the period of 2001–2007, the ecological quality of the western Langshan and Sertengshan and the eastern Damaqunshan in the Yinshan Mountains generally deteriorated, and the ecological quality of the central and eastern Damaqunshan significantly deteriorated. During the period from 2007 to 2015, the ecological quality of the Daqingshan region in the central part of the early ecological deterioration improved, while the ecological quality of most regions in the western Langshan, Sertengshan, and Wulashan was basically unchanged or improved less. During the period 2015–2023, the ecological quality of western Langshan, northern Sertengshan, and eastern Damaqunshan began to improve, and the ecological quality of central and eastern Damaqunshan and some regions of Sertengshan improved significantly (see Figure A2).

Overall, during the entire monitoring period from 2001 to 2023, the ecological quality of the central and western Langshan, Sertengshan, and Wulashan regions in the Yinshan Mountains deteriorated or deteriorated significantly to varying degrees, and the ecological quality of most regions in eastern Damaqunshan remained basically unchanged. In addition, the ecological quality of Daqingshan and some regions of Damaqunshan in the central and eastern regions improved or improved significantly to varying degrees.

To more specifically analyze the changes in ecological quality in the Yinshan Mountains from 2001 to 2023, the grade change direction of the MODIS RSEI in each period was counted, as shown in Table 5.

**TABLE 5:** Regional statistics of MODIS RSEI class type shift in the Yinshan Mountains from 2001 to 2023.

MODIS RSEI change grade	From–To	Area (km^2^)			
		2001–2007	2007–2015	2015–2023	2001–2023
−4	V → I	/	/	/	/
−3	V → II	2.50	/	0.25	0.75
	IV → I	/	/	/	/
−2	V → III	51.25	1.25	32.25	38.00
	IV → II	2366.50	24.00	36.00	278.00
	III → I	960.75	160.75	/	1268.00
−1	V → IV	835.75	74.25	1311.50	815.75
	IV → III	7845.75	1144.75	1894.75	4501.00
	III → II	**43,071.80**	5339.50	1682.50	**29,614.50**
	II → I	8563.50	11,812.30	2649.00	10,660.50
0	V → V	1611.00	1886.75	2336.50	1646.00
	IV → IV	6569.50	6654.75	8605.50	9402.25
	III → III	17,515.50	14,554.30	13,809.50	22,746.30
	II → II	9406.75	**33,497.50**	**25,075.00**	7341.25
	I → I	/	8096.75	9280.25	0.75
1	IV → V	334.50	1695.25	2418.50	2935.00
	III → IV	2112.50	5393.00	8011.00	8899.25
	II → III	125.00	8699.00	11,312.80	93.00
	I → II	2.25	1426.75	10,441.80	1.00
2	III → V	16.75	90.25	897.00	1149.25
	II → IV	1.00	832.75	1174.25	1.50
	I → III	0.20	0.75	329.75	0.75
3	II → V	/	8.25	76.75	/
	I → IV	/	/	16.50	/
4	I → V	/	/	1.50	/

*Note:* For each time period, bold values indicate the maximum change area; underlined values indicate the maximum change area excluding the no‐change grade (0).

Obviously,

(1) During 2001–2007, the region with a MODIS RSEI change grade of −1 was the largest, with the most significant type change being III → II, covering a region of 43,071.80 km^2^.
(2) During 2007–2015, the region with an unchanged MODIS RSEI grade was the largest, amounting to 64,690.05 km^2^. In the regions with a change grade of −1 during this stage, the type change II → I was more obvious, covering a region of 11812.30 km^2^.
(3) During 2015–2023, the regions with unchanged MODIS RSEI levels still maintained the largest region, at 59106.75 km^2^; among the regions with a change grade of 1, the type change II → III was more prominent, covering 11,312.80 km^2^.
(4) Over the entire monitoring period (2001–2023), the region with a MODIS RSEI change grade of −1 was the largest, with the most significant type change being III → II, covering an area of 29614.50 km^2^. Among the regions with a change grade of 1, the type change III → IV covered 8899.25 km^2^; among regions with a change grade of −2, the type change III → I covered 1268.00 km^2^; among regions with a change grade of 2, the type change III → V covered 1149.25 km^2^. From 2001 to 2023, no region with MODIS RSEI showed change grades of −4, 4, 3 and type change IV → I (MODIS RSEI change grade of −3) in the Yinshan Mountains, and the region with type change V → II was only 0.75 km^2^.

To explore the stability of ecological quality changes in the Yinshan Mountains from 2001 to 2023, based on the coefficient of variation and the natural breakpoint method, the coefficient of variation was divided into five levels of variation: level 1 (0–0.10), level 2 (0.10–0.20), level 3 (0.20–0.30), level 4 (0.30–0.40), and level 5 (0.40–1) (the spatial distribution of the coefficient of variation in each stage is shown in Figure A3).

**FIGURE 6:**
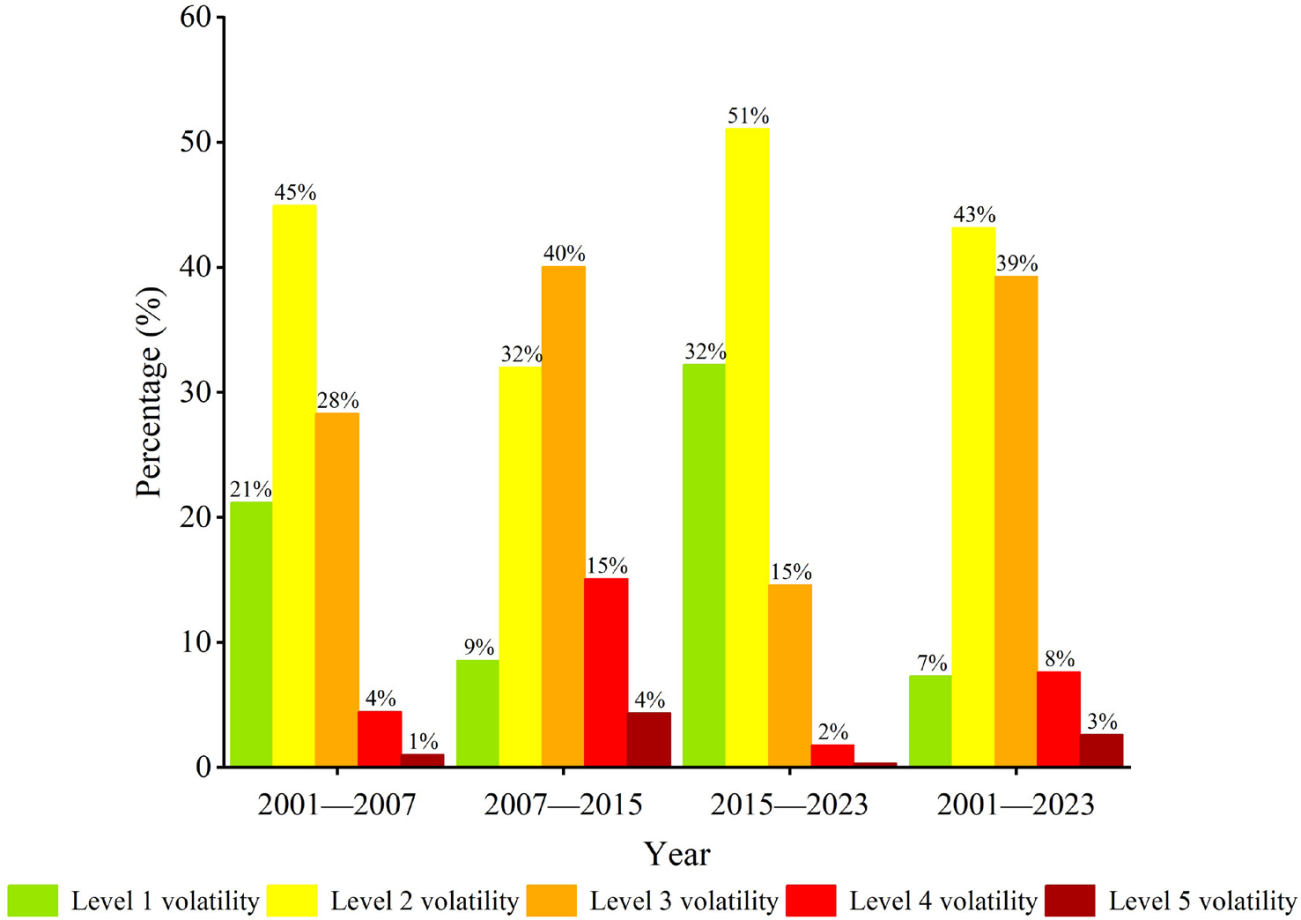
Percentage rank of MODIS RSEI coefficient of variation in the Yinshan Mountains from 2001 to 2023.

(1) In conjunction with Figure 6, from 2001 to 2007, 66.17% of the MODIS RSEI variation coefficients belonged to level 1–2 volatilities, 28.32% to level 3 volatility, and 5.51% belonged to level 4–5 volatilities, which were more drastic and mainly distributed in parts of western Langshan.
(2) From 2007 to 2015, the total proportion of MODIS RSEI variation coefficients of levels 1–2 was 41%, level 3 volatility accounted for 40.60%, and levels 4–5 accounted for 19.40% and were mainly distributed in the western Langshan and part of the eastern Damaqunshan.
(3) In 2015–2023, the MODIS RSEI values were less volatile, and the proportion of regions with coefficients of variation of 1–2 was 83.28%, which was an increase of 42.73% compared to the proportion of regions with coefficients of variation of the MODIS RSEI of 1–2 in 2007–2015.
(4) Overall, the MODIS RSEI coefficients of variation for 2001–2023 were 2–3 in the vast majority of the regions and 4–5 in only 10.28% of the regions. The distribution of the MODIS RSEI was characterized by “higher in the west and lower in the east,” and the overall environmental changes in the Yinshan Mountains were the most drastic from 2007 to 2015.

**FIGURE 7:**
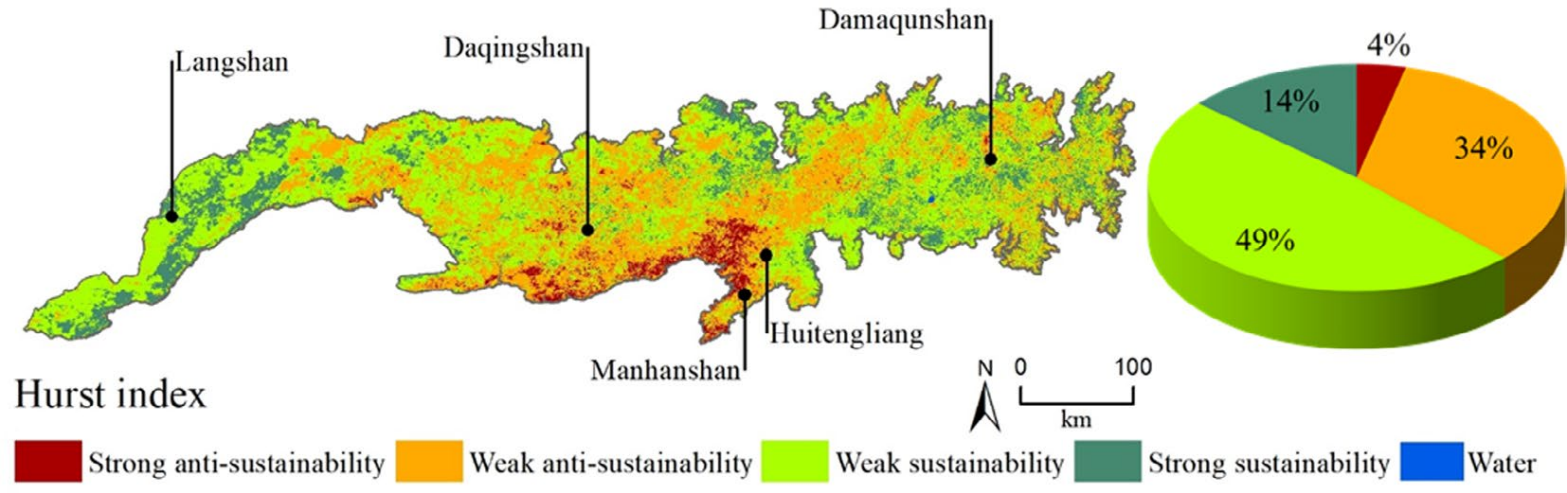
The Hurst index distribution of the MODIS RSEI in the Yinshan Mountains from 2001 to 2023.

Based on the existing time series of ecological environment changes in the Yinshan Mountains, analyzing future development trends can help identify potential problems. The Hurst (*H*) index was used to study the intrinsic trend characteristics of ecological quality and the stability of future changes in the Yinshan Mountains (Figure 7). The results showed that the *H* index of MODIS RSEI in the Yinshan Mountains was between 0.12 and 0.98, and the average value was 0.53. The closer the *H* index is to 0.50, the weaker the correlation between the sustainability and antisustainability of future changes and the past change trend; the *H* index results are set to four levels: strong antisustainability (*H* < 0.35), weak antisustainability (0.35 ≤ *H* < 0.50), weak sustainability (0.50 ≤ *H* < 0.65), and strong sustainability (*H* ≥ 0.65). The closer the *H* index is to 1, the stronger the persistence, that is, the more stable the future trend; the smaller the *H* index, the stronger the antisustainability, that is, the more unstable the future trend.

The distribution of the *H* index in the Yinshan Mountains was mainly between 0.35 and 0.65. Among them, the regions with *H* > 0.50 accounted for 62.53%, which is mainly distributed in Damaqunshan east of the Yinshan Mountains and Langshan west. The change trend of the MODIS RSEI in these regions in the coming period was the same as that in 2001–2023, with 13.68% in strong sustainability regions and 48.85% in weak sustainability regions. On the other hand, the proportion of regions with *H* < 0.50 was 37.47%, mainly distributed in the central part of the Yinshan Mountains around Daqingshan, Huitengliang, and Manhanshan, especially in the south‐central part of the region. The trend of MODIS RSEI in these regions in the future may be opposite to that in 2001–2023, with weak antisustainability regions accounting for 33.66% and strong antisustainability regions accounting for 3.81%.

## 4 Discussion

### 4.1 Analysis of Driving Factors

**FIGURE 8:**
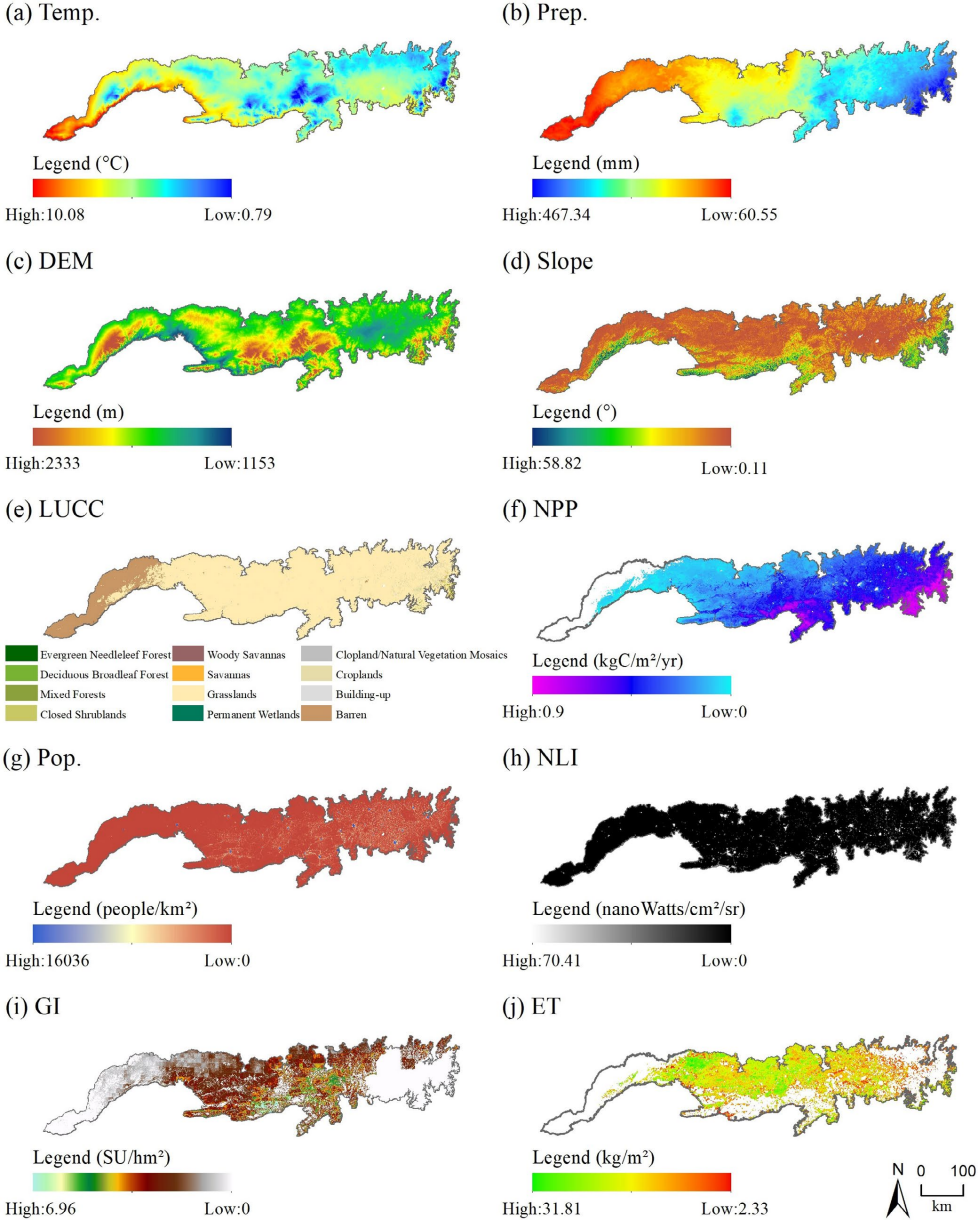
Distribution of the drivers in the Yinshan Mountains in 2023.

To obtain the driving factors of MODIS RSEI distribution and ecological quality change in the Yinshan Mountains, 10 natural and anthropogenic factors, including temperature (Temp.), precipitation (Prep.), DEM, slope, land use/cover change (LUCC), NPP, population density (Pop.), nighttime light intensity (NLI), grazing intensity (GI), and evapotranspiration (ET), were selected in this study. The OPGD model was used to analyze its influence on the spatial differentiation of MODIS RSEI. The data of each driving factor were resampled to 500 m resolution by the cubic convolution interpolation method to ensure the consistency (Figure 8).

The OPGD model reveals the implicit spatial heterogeneity formation mechanism by quantifying the explanatory power (*q* value) of each factor to the spatial differentiation of MODIS RSEI and the interaction between different factors. In this study, five discretization methods (equal interval method, natural breakpoint method, quantile method, geometric interval method, and standard deviation method) were used to optimize the parameters of the 9 continuous variables, and the number of discontinuities in the other classification methods was set to 5–10. Based on the 1 km × 1 km grid unit (a total of 26,624 sample points), the driving analysis is carried out with the data of 2023 as the representative period. The model realizes the discretization of continuous variables by optimizing the parameter combination with the highest *q* value, so as to accurately identify the dominant driving factors and their interaction effects.

**FIGURE 9:**
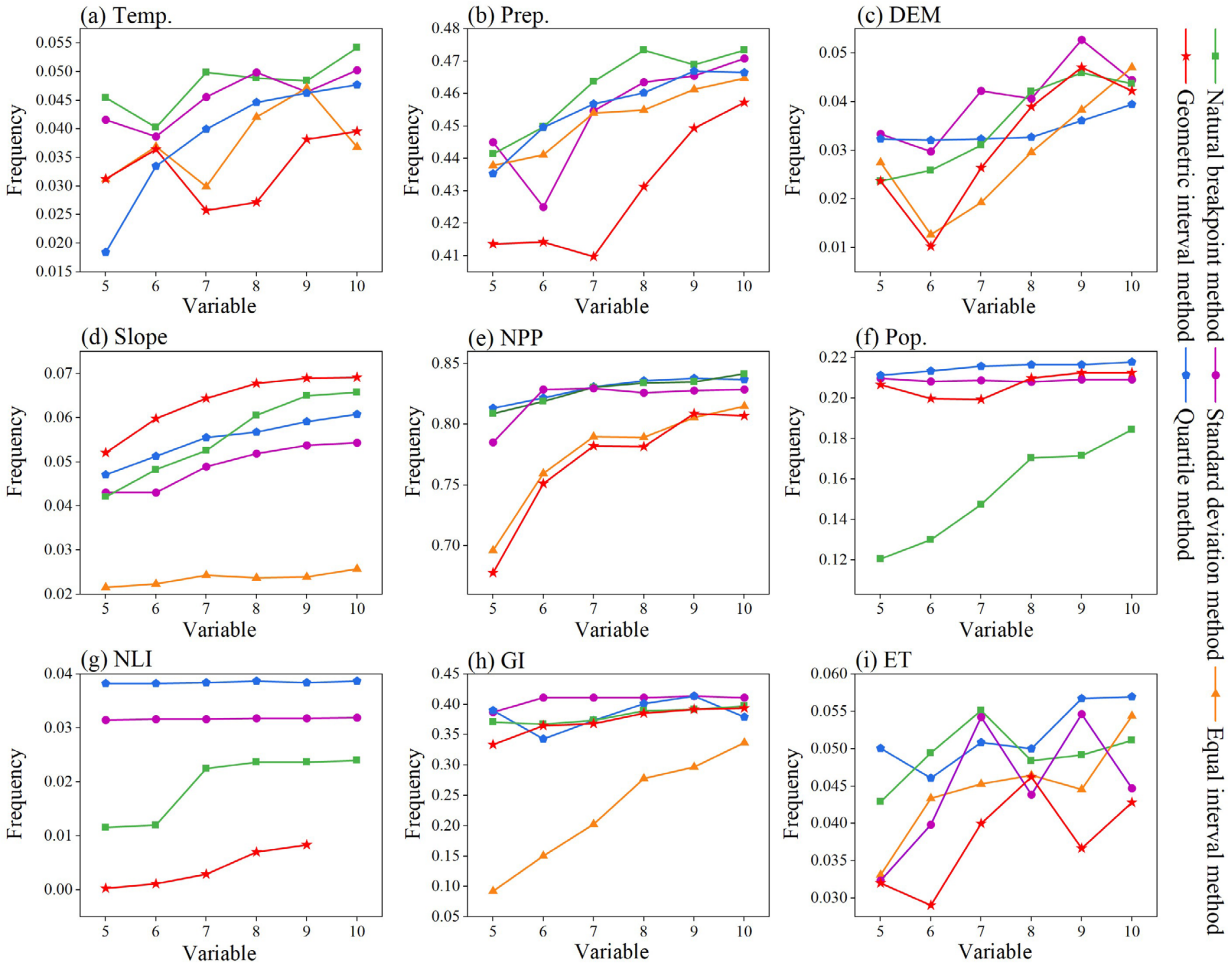
Variable discrete division of continuous variables based on the OPGD model.

**FIGURE 10:**
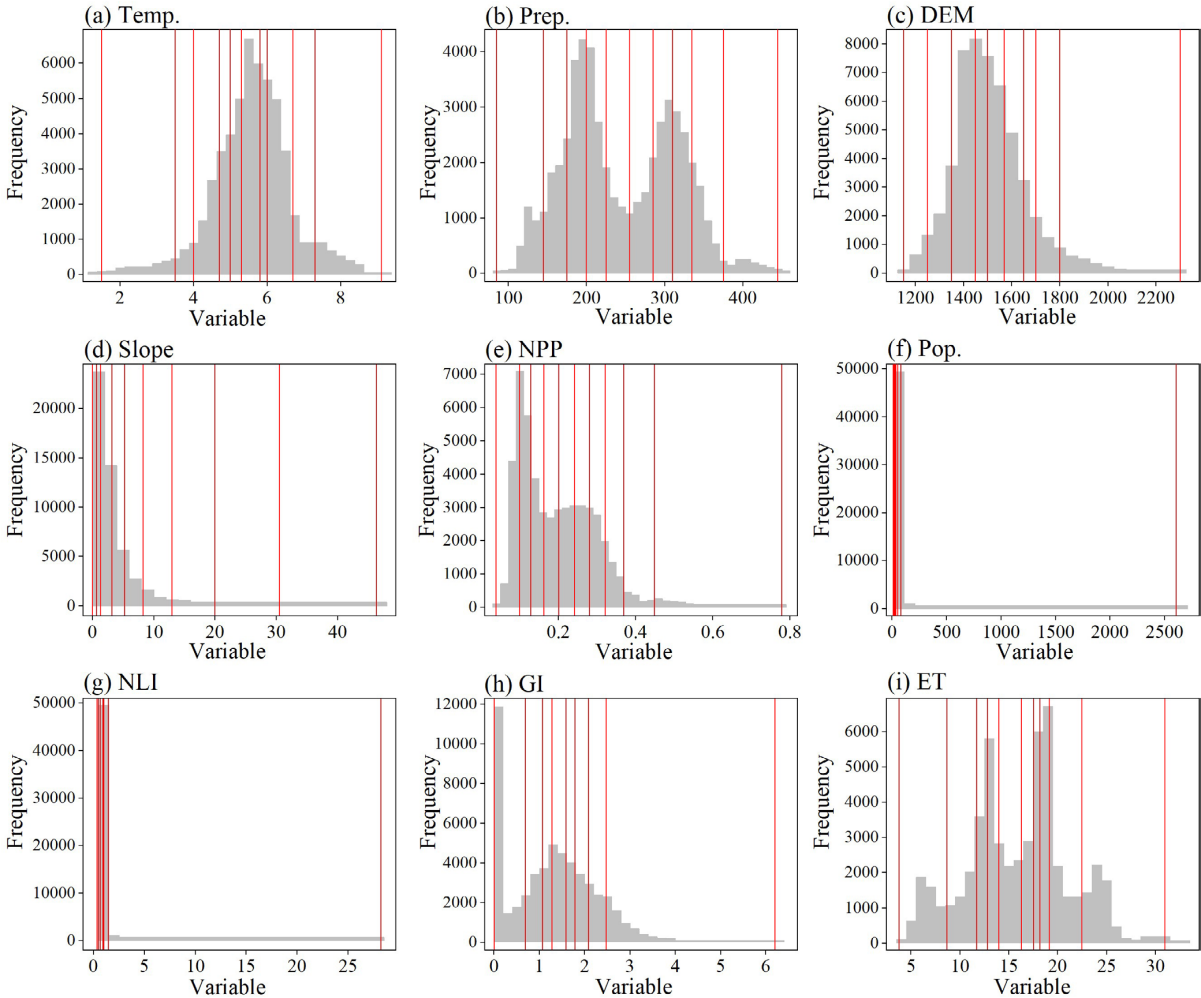
Partitioning results for continuous variables based on the OPGD model.

Based on the OPGD model, this study obtained the partition results of continuous variables and the detection results of variable discrete partitions (Figures 9 and 10). By analyzing the *q* values of different discretization methods, it is found that when the *q* value reaches the peak, the corresponding discrete parameter combination is the optimal solution. Specifically, the optimal discrete parameter combination of Temp., Prep. and NPP is the natural breakpoint method with 10 discontinuities; the optimal discrete parameter combination of Pop. and ET is the quantile method of 10 discontinuities. The optimal discrete parameter combination of DEM is the standard deviation method of 9 discontinuous numbers. The optimal discrete parameter combination of slope is the geometric interval method of 10 discontinuous numbers. The optimal discrete parameter combination of NLI is the quantile method of 8 discontinuous numbers. The optimal discrete parameter combination of GI is the quantile method of 9 discontinuous numbers (Figure 9). Therefore, for different continuous variables, there may be significant differences in the combination of discrete methods and number of discontinuities (Figure 10). This difference reflects the uniqueness of the driving factors in spatial distribution and ecological impact mechanisms. By selecting the optimal discrete parameter combination, the OPGD model can more accurately reveal the explanatory power of the driving factors on the spatial differentiation of ecological quality, thus providing a scientific basis for the analysis of the driving mechanism of ecological quality changes.

**FIGURE 11:**
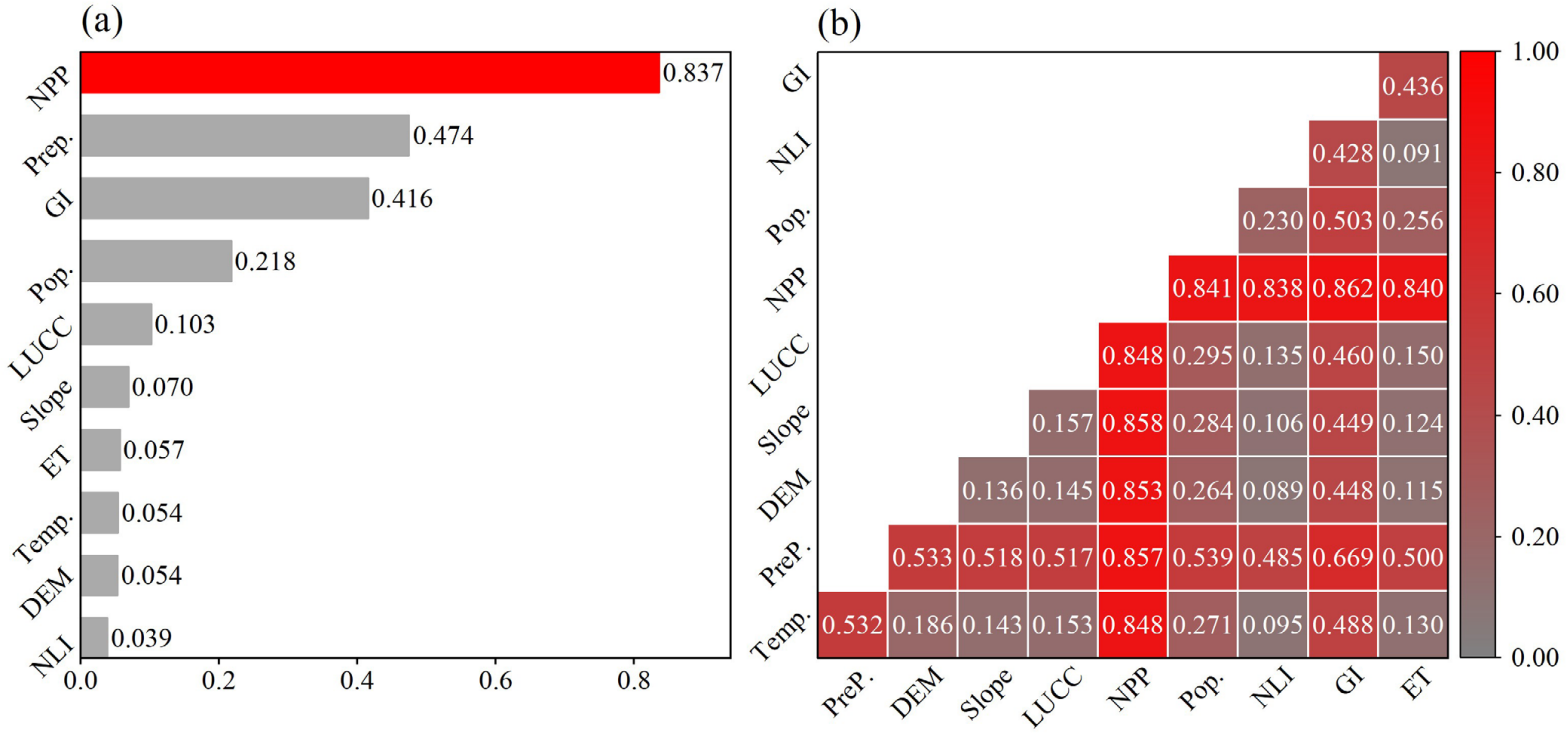
Driving factor analysis based on OPGD model. (a) Effect of a single factor on changes in the MODIS RSEI (*q* value); (b) Driver interaction results (*q* value).

Based on the OPGD model, single‐factor and multifactor interactive detection of the contribution of Temp., Prep., DEM, slope, LUCC, NPP, Pop., NLI, GI, and ET to MODIS RSEI was further carried out (Figure 11).

#### 4.1.1 Factor Detection Results

Factor detection is used to quantify the impact of various driving factors on the ecological quality (MODIS RSEI). The larger the *q* value, the stronger is the explanatory power of the driving factor of ecological quality (MODIS RSEI). Figure 11a shows the contribution of Temp., Prep., DEM, slope, LUCC, NPP, Pop., NLI, GI, and ET to MODIS RSEI. Each driving factor has a significant effect on the MODIS RSEI changes in the Yinshan Mountains (*p* < 0.01). The descending order of *q* values is as follows: NPP > Prep. > GI > Pop. > LUCC > slope > ET > Temp. > DEM > NLI.

Among them, NPP, Prep., and GI are the main drivers affecting the MODIS RSEI changes in the Yinshan Mountains, with *q* values all above 0.410, indicating strong explanatory power. NPP, as a direct reflection of vegetation productivity, determines the energy and material basis of the ecosystem through its spatial pattern; Prep., as a key hydrothermal condition, directly regulates the degree of water stress for vegetation in arid and semiarid regions, while grazing intensity represents the most common form of human disturbance in the region, directly affecting ecosystem health by altering vegetation structure and soil physical properties. In contrast, NLI, DEM, and Temp. have relatively smaller effects on ecological quality, with *q* values below 0.055. This is mainly because, at the regional scale, the direct ecological effects of these factors are often masked or regulated by stronger climatic processes (such as precipitation patterns) and human activities (such as grazing and land use). Particularly for NLI, its indicative range in the Yinshan Mountains, which is not highly urbanized, is limited, reflecting mainly the distribution of point‐like towns, while having a weak direct impact on the primary ecological components such as vast grasslands and forests.

#### 4.1.2 Interaction Detection Results

Interaction detection is used to assess whether the interaction of two driving factors on ecological quality (MODIS RSEI) is enhanced, weakened, or independent. As shown in Figure 11b, the interaction of any two driving factors is greater than the influence of a single driving factor on the spatial differentiation of MODIS RSEI in the Yinshan Mountains. The interaction types are mainly two‐factor or nonlinear enhancement, indicating that the spatial differentiation of MODIS RSEI in the Yinshan Mountains is not caused by a single influencing factor, but the result of multiple factors.

Among them, the interaction between NPP and GI has the strongest impact on the spatial differentiation of MODIS RSEI in the Yinshan Mountains, with a *q* value of 0.862, indicating an influence of nearly 90%. This is mainly because NPP, as a direct reflection of vegetation productivity, and grazing, as a direct human intervention, act jointly: in areas with high NPP, excessive grazing can weaken vegetation resilience, while in areas with low NPP, which are inherently ecologically vulnerable, grazing activities further exacerbate degradation. It is this coupling of natural productivity and human disturbance that collectively shapes the final pattern of regional ecological quality. In addition, the interaction between NPP and other driving factors also shows strong influence, with *q* values all greater than 0.830. Specifically: NPP ∩ GI (*q* = 0.862) > NPP ∩ slope (*q* = 0.858) = NPP ∩ Prep. (*q* = 0.857) > NPP ∩ DEM (*q* = 0.853) > NPP ∩ Temp. (*q* = 0.848) = NPP ∩ LUCC (*q* = 0.848) > NPP ∩ Pop. (*q* = 0.841) > NPP ∩ ET (*q* = 0.840) > NPP ∩ NLI (*q* = 0.838). These results indicate that the interaction of NPP with topography, land cover, climate, and socio‐economic factors has an important impact on the spatial differentiation of ecological quality.

On the other hand, the interaction between Prep. and the other driving factors also has a notable influence, with *q* values all greater than 0.480. Specifically: Prep. ∩ GI (*q* = 0.669) > Prep. ∩ Pop. (*q* = 0.539) = Prep. ∩ DEM (*q* = 0.533) > Prep. ∩ Temp. (*q* = 0.532) > Prep. ∩ slope (*q* = 0.518) > Prep. ∩ LUCC (*q* = 0.517) > Prep. ∩ ET (*q* = 0.500) > Prep. ∩ NLI (*q* = 0.485). These results indicate that the interaction of Prep. with topography, land use, climate, and socio‐economic factors also contributes significantly to the spatial differentiation of ecological quality.

In contrast, the interactions of ET and NLI with other factors have relatively low influence, consistent with the results of single‐factor detection. The low *q* values of the ET and NLI indicate that they are not dominant independent driving forces at the regional scale. As a key link in water‐energy exchange, the spatial variation of evapotranspiration is largely determined by core climatic and biological factors such as Prep. and NPP. NLI, as a proxy for human activity, has a relatively limited direct impact in regions dominated by natural ecological backgrounds like the Yinshan Mountains, making it difficult to form strong synergistic or antagonistic effects with other factors.

It is worth noting that factors with weak explanatory power in single‐factor detection (such as DEM and NLI) see a significant improvement in their explanatory power when interacting with dominant factors (such as NPP and GI). Taking DEM as an example, its single‐factor *q* value is only 0.054, but after interacting with NPP, the *q* value reaches 0.853, an increase of more than 15 times; the single‐factor *q* value of NLI is 0.039, which rises to 0.838 after interaction with NPP, an increase of over 21 times. This phenomenon of “weak factor reinforcement” further confirms the multifactor coupling characteristics of regional ecological quality formation mechanisms.

These findings indicate that when formulating ecological protection and restoration strategies, attention should not only be paid to the independent effects of dominant factors such as NPP, Prep., and GI but also to the interactions between various factors. In particular, emphasis should be placed on factor combinations that have relatively weak individual effects but can significantly enhance overall explanatory power through interaction, adopting a systematic and comprehensive governance approach.

### 4.2 Performance Verification of MODIS RSEI Model

#### 4.2.1 MCD12QI Data Were Introduced to Verify the Performance Difference Between the RSEI and MODIS RSEI Models

**FIGURE 12:**
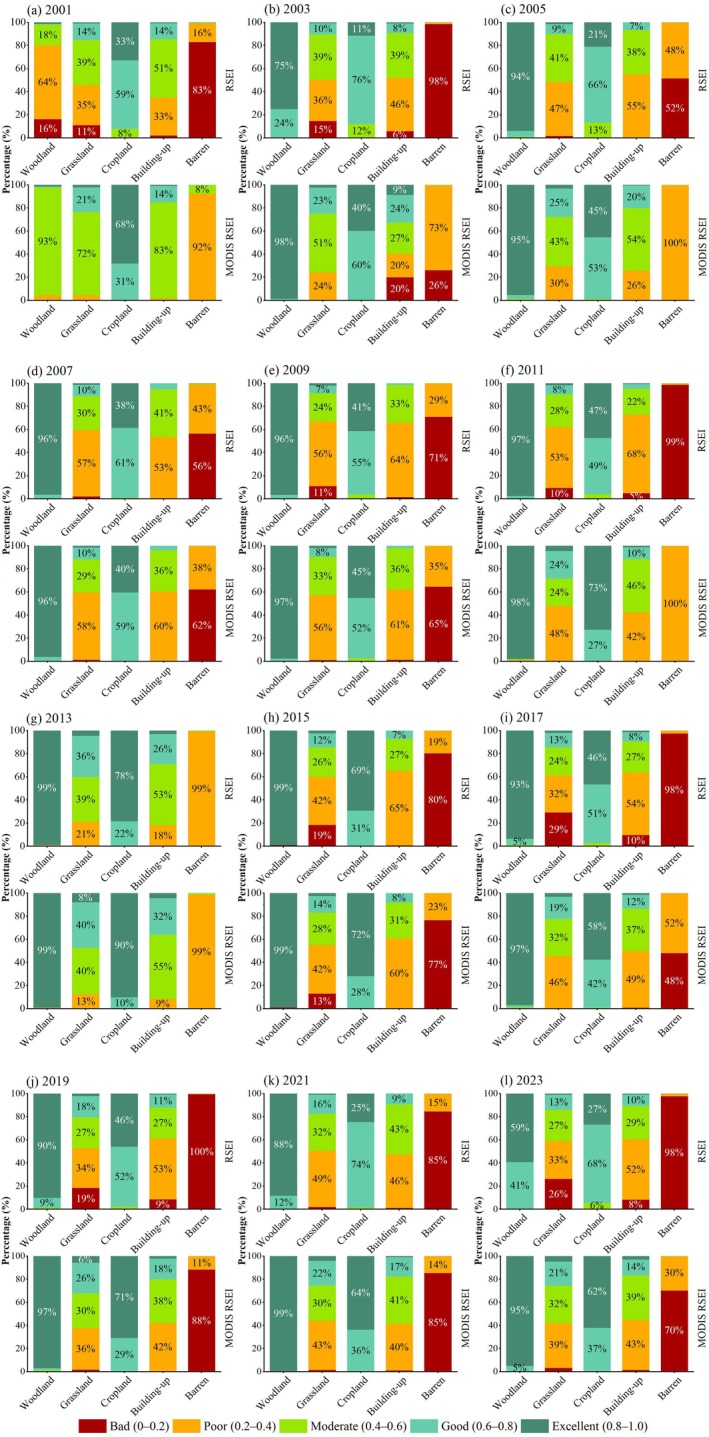
Proportions of different ecological levels of RSEI and MODIS RSEI in different land covers.

By comparing the spatial distribution of RSEI (Figure S1) and MODIS RSEI (Figure 5) in the Yinshan Mountains from 2001 to 2023, the difference analysis (Figure S2) shows that the difference Δ_(MODIS RSEI‐RSEI)_ between MODIS RSEI and RSEI in the study area is mainly positive, which means that the improved model is more optimistic about the overall ecological quality of the region. In order to further explore the performance differences between models, the ecological quality grade distribution under different land cover types was further analyzed. The study selected 12 MCD12Q1 v6.1 datasets from 2001 to 2023, corresponding to the time series of this study. In this dataset, the land cover of the region was classified into five types: woodland, grassland, farmland, construction land, and bare land. In theory, the richer the vegetation, the better the ecological quality evaluation results based on the RSEI and MODIS RSEI, and vice versa. The ecological quality (RSEI and MODIS RSEI) levels for the different land cover types are shown in Figure 12.

For different land cover types, the distribution of RSEI levels showed significant differences: the RSEI levels of woodland and cropland were mainly “excellent” and “good,” the RSEI levels of grassland and construction land were mainly “moderate” and “poor,” and the RSEI levels of barren were mainly “bad” and “poor.” Similarly, the MODIS RSEI grade distribution also showed a similar trend: the MODIS RSEI grades of woodland and cropland were mainly “excellent” and “good,” the MODIS RSEI grades of grassland and architectural land were mainly “moderate” and “poor,” and the MODIS RSEI grades of barren were mainly “bad” and “poor.” At the same time, it can be seen that the ecological rating of MODIS RSEI is higher than that of RSEI in both high vegetation coverage area and low vegetation coverage area. This is because MODIS RSEI is optimized based on RSEI, which can better overcome the influence of the soil background in arid and semiarid regions, improve the accuracy of ecological ratings, and provide a more reliable tool for ecological quality assessments in arid and semiarid regions.

#### 4.2.2 Global High‐Resolution (1 km × 1 km) Soil Salinity Data Was Introduced to Verify the Difference in Performance Between the RSEI and MODIS RSEI Models

Soil salinity is the core parameter to characterize soil quality and health status, and it is also one of the most important stress factors in arid and semiarid ecosystems. As a key indicator of land degradation and ecological vulnerability, soil salinity is highly sensitive to regional water and heat balance and human activities, which can effectively indicate the level of ecological stress in the region. Therefore, the performance difference between RSEI and MODIS RSEI can be verified by comparing the correlation between the two models and soil salinity.

Based on the data analysis in Table 6, it can be seen that over the long time series from 2001 to 2023, except for the year 2001, the negative correlation between MODIS RSEI and soil salinity was stronger than that of RSEI in all other years (numbers highlighted in Table 6). Specifically, the absolute value of the correlation coefficient between MODIS RSEI and salinity increased significantly in most years, for example, from −0.52 to −0.55 in 2005 (5.77%), from −0.67 to −0.69 in 2021 (2.99%), and from −0.57 to −0.61 in 2023 (7.02%). From the time series average perspective, the mean correlation coefficient between MODIS RSEI and salinity (−0.49) is approximately 4.30% higher than that of RSEI (−0.47), indicating that MODIS RSEI has a more stable and stronger capability in capturing salinity stress signals. This result confirms that by introducing indices optimized for characteristics of arid and semiarid regions (such as salinity index), MODIS RSEI can respond more sensitively to soil salinization, a key ecological stress factor, compared to the traditional RSEI. Therefore, in the ecological quality assessment of arid and semiarid regions, MODIS RSEI demonstrates superior overall performance, particularly showing significant advantages in monitoring and evaluating salinity‐driven ecological degradation.

**TABLE 6:** Comparison of correlation coefficients between RSEI, MODIS RSEI, and salinity.

Year	The correlation coefficient between RSEI and salinity	The correlation coefficient between MODIS RSEI and salinity	Absolute value increase percentage
2001	−0.46	−0.44	−4.35%
2003	−0.50	**−0.52**	**4.00%**
2005	−0.52	**−0.55**	**5.77%**
2007	−0.30	**−0.33**	**10.00%**
2009	−0.40	**−0.41**	**2.50%**
2011	−0.48	**−0.51**	**6.25%**
2013	−0.53	**−0.55**	**3.78%**
2015	−0.30	**−0.32**	**6.67%**
2017	−0.40	**−0.43**	**7.50%**
2019	−0.45	**−0.48**	**6.67%**
2021	−0.67	**−0.69**	**2.99%**
2023	−0.57	**−0.61**	**7.02%**
Mean	−0.47	**−0.49**	**4.90%**

*Note:* Bold formatting in the MODIS RSEI column denotes stronger absolute salinity correlation relative to RSEI, while in the percentage column it signifies positive growth.

#### 4.2.3 High‐Resolution (1 km × 1 km) Ecological Environment Quality Data of China (CHEQ, 2001–2019) Were Introduced to Verify the Difference in Performance Between the RSEI and MODIS RSEI Models

**FIGURE 13:**
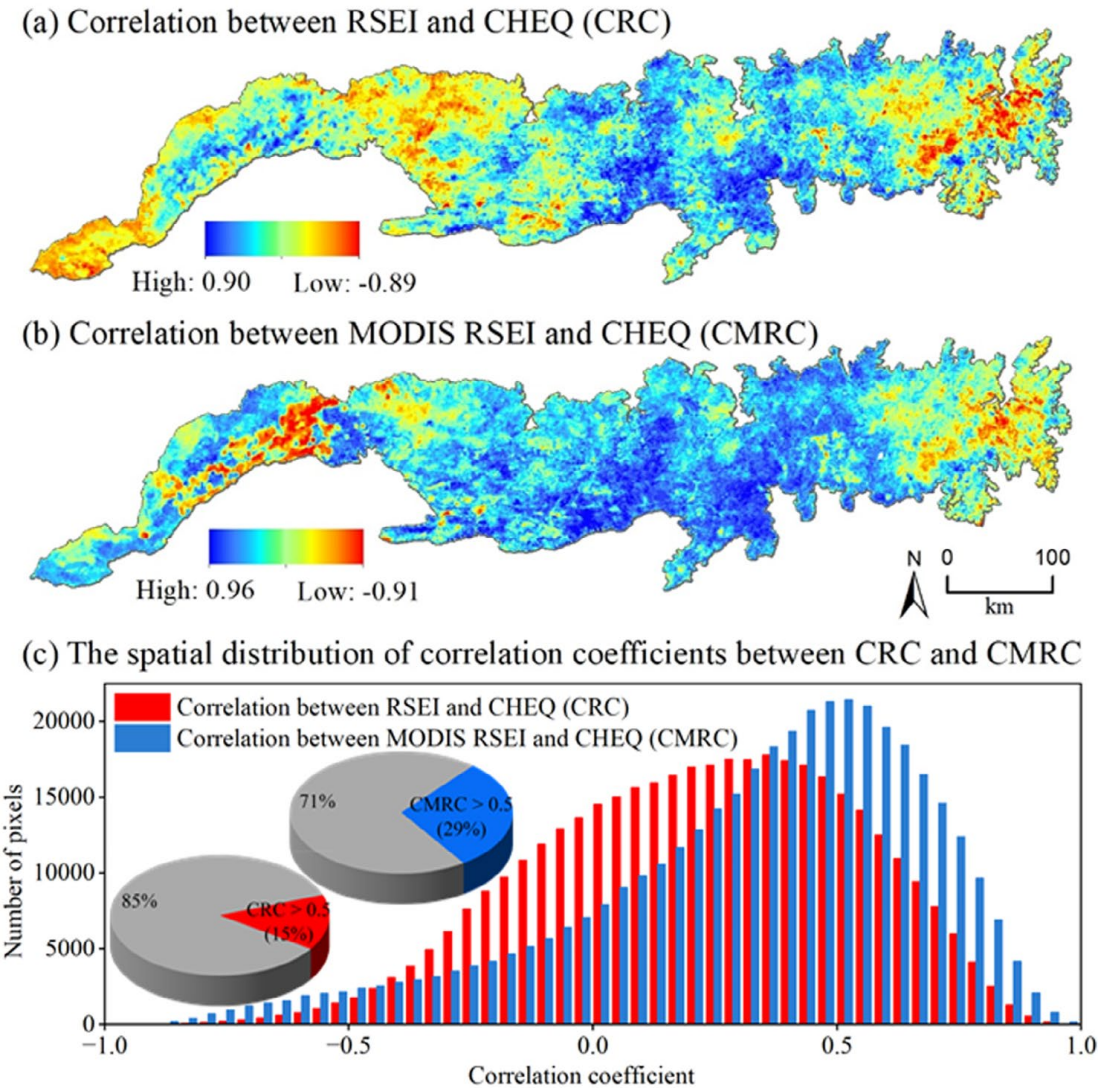
The correlation coefficient and spatial distribution of RSEI, MODIS RSEI, and CHEQ.

To better verify the reliability of MODIS RSEI, this study compared and evaluated the performance of MODIS RSEI and RSEI based on the third‐party ecological data CHEQ. The results show that the correlation between MODIS RSEI and CHEQ (CMRC) is significantly better than that of RSEI (CRC) in both numerical and spatial distribution (Figure 13): (1) CMRC not only has a higher upper limit (0.96 > 0.90), but also its proportion of significant positive correlation pixels (29.46%) is nearly twice that of CRC (15.01%); (2) the distribution of CMRC values is more concentrated in the high correlation interval, indicating that it has a more stable and consistent high correlation with CHEQ in the whole region. In summary, MODIS RSEI has higher accuracy and reliability in characterizing the spatial pattern of ecological quality, and the model optimization is successful.

### 4.3 Comparison With Similar Studies

#### 4.3.1 Comparison With Studies in the Same Type of Desertified Steppes

Research on the evolution of desertified steppe ecosystems in arid and semiarid regions has mostly focused on the correlation between land cover change and single driving factors (Da Silva et al. 2023; Jargalsaikhan et al. 2024; Na et al. 2021; Villarreal et al. 2016; Wu, Hao, et al. 2024; Zhao, Wang, et al. 2023). By combining the recent literature, it is found that the existing research presents the following characteristics in methodology and index selection:

##### 4.3.1.1 Limitations of Time Scale

Most studies used images at the beginning and end of the study period or at short‐term intervals. Although they can capture periodic changes, it is difficult to reveal the long‐term continuous evolution (Jin et al. 2023; Li, Liu, et al. 2024). Su et al. (2022) studied the process of litter decomposition in alpine meadows in the Tianshan Mountains of Northwest China from 2019 to 2021, revealing the dependence of litter decomposition on litter quality, climate change, and grassland types. Based on observational data in 2010, 2015, and 2018, Zhang, Gu, et al. (2024) analyzed the multitemporal characteristics and spatial changes in soil wind erosion in nine sub‐basins of the Tarim River Basin using a soil wind erosion model customized for cultivated land, grassland, and desert topography. Selka et al. (2024) used the Google Earth Engine (GEE) to evaluate the impact of land cover change on surface temperature in the semiarid region of northwestern Algeria during 1989–2019 (1989, 1999, 2009, and 2019). Although these studies provide insights into staged ecological changes, it is difficult to fully reflect the long‐term dynamic response of the ecosystem because of the limited temporal resolution of the data.

##### 4.3.1.2 Singleness of Indicators

Eighty percent of the studies relied on a single index or a few static parameters and lacked multifactor coupling analysis (Du et al. 2021; Noojipady et al. 2015). For example, Li et al. (2021) evaluated the ecological impact of the transition from desertified steppe to shrub land by analyzing soil nitrogen content but ignored the changes in other nutrients, such as soil carbon and phosphorus, as well as key factors, such as microbial activity, soil structure, and water dynamics, which may lead to insufficient understanding of the overall impact of ecosystem transformation. Zoungrana et al. (2024) predicted grassland coverage in the Burkina Faso steppe of Africa based on MODIS NDVI but did not integrate soil moisture or salinization indicators, which may underestimate the complexity of ecological quality in arid regions. Zhou et al. (2024) evaluated ecological quality by analyzing the changes in grassland NPP (MODIS) in the Qinghai‐Tibet Plateau but did not fully consider other key factors such as soil quality, species diversity, and land use change, resulting in insufficient explanatory power for ecosystem complexity. The limitations of these studies in the selection of indicators highlight the importance of multifactor coupling analysis in ecological quality assessment.

##### 4.3.1.3 The Method Is Not Universal Enough

The region‐specific model has limitations for cross‐regional promotion (Bacar and Faque 2024; Zhang et al. 2022). For example, Cao et al. (2023) designed a soil wind erosion model RWEQ (Revised Wind Erosion Equation) for the Qaidam Basin but did not verify its applicability in other arid regions (such as the Yinshan Mountains). In addition, there are significant differences in the methodology and indicator selection among different studies. For example, Muraina et al. (2023) used overlay analysis to assess grassland coverage (MODIS MCD12Q1), while Dey et al. (2024) used the analytic hierarchy process and multicriteria decision‐making (MCDM) tools to assess grassland degradation. These differences reflect the diversity and complexity of the ecological quality assessment of desertified steppes and highlight the necessity of developing universal models.

#### 4.3.2 Comparison With Studies in the Yinshan Mountains

The Yinshan Mountains has bred a desertified steppe landscape because of its unique natural conditions. However, existing studies have focused more on local ecological characteristics. For example:

Yang and Wang (2019) studied the effects of land use pattern on climate and ecosystem services in the northern foot of the Yinshan Mountains. Han et al. (2024) explored the effects of forage intercropping and nitrogen application on soil characteristics and forage yield at the southern foot of the Yinshan Mountains in Inner Mongolia. Yuchi et al. (2021) analyzed the relationship between meteorological factors and drought index PA value by monitoring the precipitation changes at different slope positions in the northern foot of the Yinshan Mountains. Although these studies have revealed some ecological characteristics of the Yinshan Mountains, they are limited by the singleness of the research scope or indicators and do not fully reflect the overall ecological quality of the area.

In addition, Luo (2023) studied the community classification of desertified steppe in the Yinshan Mountains and analyzed the horizontal and vertical distribution patterns of plant communities in this area over 1 year. Yan et al. (2025) used PKU GIMMS NDVI as a vegetation growth index to explore the spatial and temporal heterogeneity of vegetation and its driving mechanisms in desertified steppe ecosystems in arid and semiarid regions. According to the results of the third national census of traditional Chinese medicine resources, Zhang et al. (2020) counted the species of medicinal plant resources in 31 counties around the Yinshan Mountains and analyzed the differences in the spatial distribution of medicinal plant resources in the Yinshan Mountains of Inner Mongolia. In general, although these studies have made important progress in ecological research in the specific Yinshan Mountains, it is difficult to fully reflect the long‐term evolutionary characteristics and driving mechanisms of ecological quality in the Yinshan Mountains because of the short time span and single index.

To comprehensively analyze the evolutionary characteristics of ecological quality in the Yinshan Mountains, Xing et al. (2024) used Landsat remote sensing images, coupled with five indicators of greenness (NDVI), humidity (WET), dryness (NDBSI), Topsoil Grain Size Index (TGSI), and heat (LST) to construct an improved remote sensing ecological index TRSEI, and evaluated the ecological quality of the Yinshan Mountains. Although the TRSEI model has made progress in soil moisture conservation in arid and semiarid regions, it has obvious deficiencies in the salinity index, which reflects the degree of soil salinization. In addition, the model failed to fully consider the impact of sparse vegetation and the soil background on the index, resulting in limited applicability in areas with low vegetation coverage. At the same time, Landsat images are limited by cloud cover and the satellite revisit cycle, which further affects the accuracy and reliability of the TRSEI. These limitations indicate that TRSEI still has room for improvement in ecological quality assessment in arid and semiarid regions.

In summary, this study constructed a comprehensive ecological index MODIS RSEI based on MODIS data, combined with greenness (SAVI), humidity (SWCI), dryness (NDBBI), heat (LST), and comprehensive salinity index (CSI), to comprehensively and objectively evaluate the ecological quality change trend of the Yinshan Mountains from 2001 to 2023 and to detect the response of natural and human factors to the ecological quality of the Yinshan Mountains. Compared with TRSEI, MODIS RSEI performs better in salinization monitoring, soil background correction, and data continuity, and can more accurately reflect the long‐term evolution characteristics of ecological quality in the Yinshan Mountains.

### 4.4 Limitations of the Study

Based on the RSEI, this study systematically optimized and constructed the MODIS comprehensive ecological index MODIS RSEI, which effectively solved the applicability of the RSEI in the ecological quality evaluation of arid and semiarid regions, further enriches the index evaluation system of the region, and provides a new research idea for regional ecological quality evaluation. However, there are still some limitations and scope for future research.

(1) Limitations of the spatiotemporal resolution of data sources and processing workflow. Although the MODIS data used in the study overcome the limitations of Landsat data affected by cloud cover and satellite revisit periods, their relatively low spatial resolution and 8‐day temporal interval restrict the model's evaluation accuracy in complex underlying surface areas and its ability to capture short‐term ecological disturbances. In addition, the current data preprocessing and computational workflows are not highly automated, which affects research efficiency and the timeliness of results.
(2) Optimization potential of core model parameters and evaluation systems. In model construction, the SAVI uses a fixed soil adjustment factor *L*, which lacks adaptability in complex geographic environments. Meanwhile, masking water bodies to avoid interference prevents the assessment of water quality, which has important ecological functions. Future work should dynamically optimize key parameters and systematically incorporate water body ecological quality into the overall evaluation framework.
(3) Insufficient consideration of external driving factors in future trend prediction. Predictions based on the Hurst index mainly reflect the continuation of historical trends and do not fully account for key external drivers such as uncertainties due to climate change and policy interventions. Subsequent studies should couple climate scenario simulations with policy effect assessments to improve the scientific reliability of predictions and their value for decision‐making.

## 5 Conclusions and Suggestion

In this study, based on MODIS data, a MODIS RSEI ecological quality evaluation model suitable for arid and semiarid regions was constructed. Taking the Yinshan Mountains as a typical case, the spatial and temporal evolution law and driving mechanism of ecological quality from 2001 to 2023 were systematically evaluated. Compared with the traditional RSEI model, the MODIS RSEI model shows better applicability in arid and semiarid regions: (1) the use of MODIS data effectively overcomes the limitations of cloud cover and revisit period and ensures the integrity of long‐term sequence data; (2) the optimized greenness indicator (SAVI) significantly reduced the soil background interference and improved the monitoring accuracy in the low vegetation coverage area; (3) the improved soil moisture indicator (SWCI) enhances the ability to identify small changes in soil moisture; (4) the innovative dryness indicator (NDBBI) improves the identification accuracy of bare soil and urban areas; and (5) the newly added comprehensive salinity indicator (CSI) has achieved effective monitoring of soil salinization.

The main conclusions are as follows:

(1) From 2001 to 2023, the areas with significant deterioration in ecological quality in the Yinshan Mountains accounted for 1.56%, and the areas with deterioration accounted for 44.97%. Among these, there were more areas with one grade of ecological quality decline. Areas with poor and bad ecological qualities were mainly distributed west of Langshan and Sertengshan. The areas with good and excellent MODIS RSEI grades were mainly distributed east of Damaqunshan, showing a step‐by‐step distribution characteristic of “lower in the west and higher in the east”.
(2) From 2001 to 2023, the coefficient of variation of MODIS RSEI in most areas of the Yinshan Mountains was relatively low and moderate volatilities (levels 2 and 3), accounting for 43.17% and 39.27%, respectively, and the coefficient of variation of MODIS RSEI showed spatial distribution characteristics of “higher in the west and lower in the east.” The Hurst index analysis showed that the *H* index distribution in the Yinshan Mountains was mainly in the range of 0.35–0.65. The change trend of MODIS RSEI in the eastern Damaqunshan and the western Langshan and Sertengshan in the future is consistent with the change trend from 2001 to 2023, while the change trend of MODIS RSEI in the central region (especially the Daqingshan, Huitengliang, and Manhanshan in the central and southern regions) in the future may be opposite to the past.
(3) The ecological quality of the Yinshan Mountains was affected by a variety of natural and anthropogenic factors, and the contribution rate of the single‐factor detection results was significantly lower than that of multifactor interaction. The results of single‐factor detection showed that NPP, Prep., and GI had significant effects on the ecological quality of the Yinshan Mountains. The results of the multifactor interaction detection showed that the interaction of any two driving factors was greater than the influence of a single driving factor on the spatial differentiation.

The innovative contributions of this study are as follows: (1) using international standard data products and long‐term, full‐coverage medium‐resolution imaging spectrometer data, an evaluation basis with both universality and spatiotemporal details is constructed, which is not only convenient for technology promotion and horizontal comparison, but also can accurately capture the ecological evolution process of medium and large geographical units; (2) MODIS RSEI considering salinity (CSI) and greenness (SAVI), humidity (SWCI), dryness (NDBBI), and heat (LST) components is more suitable for remote sensing ecological quality assessment in arid and semiarid regions, and more accurately reveals the change trend of ecological quality, which provides theoretical support for precise regional ecological management and regional sustainable development; (3) the OPGD model is integrated to systematically identify the independent and interactive effects of natural and human factors on ecological quality, which provides a more profound insight into the driving mechanism of regional ecological evolution.

**FIGURE 14:**
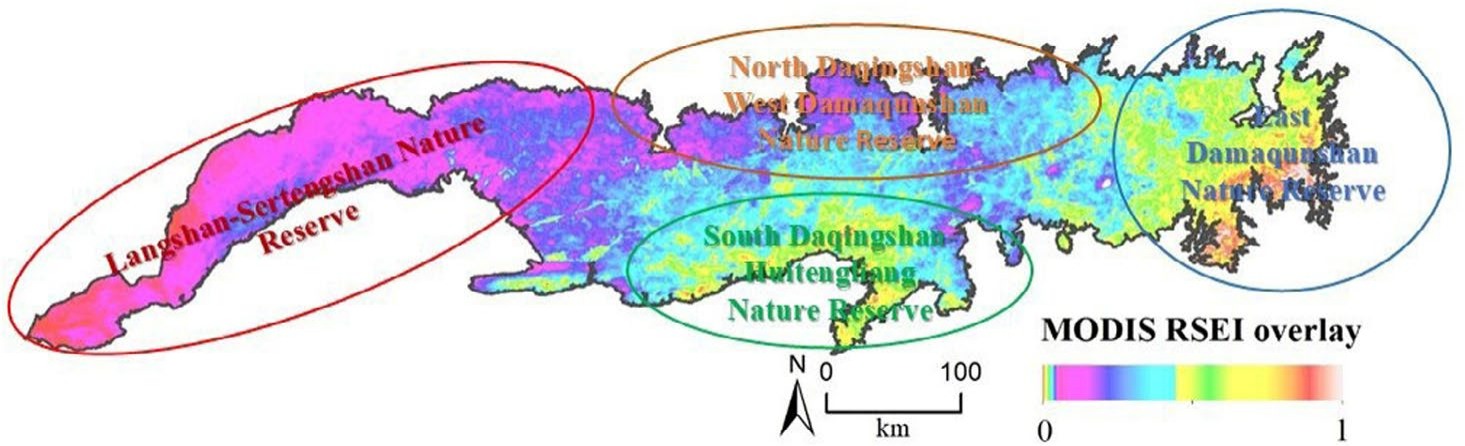
Ecological protection zoning of the Yinshan Mountains based on MODIS RSEI overlay.

Based on the above conclusions, this study puts forward the following policy recommendations for the sustainable development of the Yinshan Mountains:

(1) Implement differentiated ecological restoration and protected area construction. In the Langshan and Sertengshan where the ecological quality has significantly degraded, promote key ecological projects focused on vegetation restoration, soil and water conservation, and soil improvement, and implement rotational grazing systems and control livestock carrying capacity to curb land desertification and grassland degradation. In areas with relatively better ecological conditions, such as the eastern Damaqunshan, southern Daqingshan, and Huitengliang, establish ecological protected areas, strictly restrict development activities, prioritize the protection of native ecosystems and biodiversity, explore a “community co‐management” mechanism, and promote the synergy between protection and community development. At the same time, in transitional zones such as the western Damaqunshan and northern Daqingshan, implement an ecological conservation strategy that is “primarily natural restoration with assisted artificial interventions”, actively explore eco‐friendly industrial development models, strictly enforce the system of livestock‐to‐grass balance, encourage the development of under‐forest economies, ecotourism, and specialized sand‐related industries, and directly link ecological protection outcomes to community economic benefits (Figure 14).

(2) Optimize the spatial pattern of land and promote coordinated regional development. According to the ecological quality pattern of “low in the west, high in the east” implement land use policies in the ecologically fragile western areas that prioritize protection and restoration, strictly control agricultural and pastoral expansion, and promote the return of farmland to forests and grasslands. In ecologically favorable areas such as the eastern Damaqunshan, moderately develop low‐impact industries such as ecotourism and organic agriculture to realize the value of ecological products. It is recommended to develop a coordinated planning strategy for ecological protection and industrial development in the Yinshan Mountains, clarify functional zoning, and achieve resource‐adaptive utilization.
(3) Strengthen climate adaptation capacity and carbon sequestration value transformation. Establish and improve a climate change monitoring and early warning system in the Yinshan Mountains, and formulate agricultural adjustment strategies to adapt to climate change. For instance, promote water‐saving irrigation and drought‐resistant crop varieties in areas with highly variable precipitation. At the same time, explore regional carbon sequestration compensation mechanisms, incorporate ecological restoration projects in the Yinshan Mountains (such as vegetation restoration in Langshan and forest conservation in Daqingshan) into the carbon trading system, promote market‐oriented operation of carbon projects, and attract social funds to participate in ecological construction.
(4) Promote green industry transformation and population‐resource optimization regulation. Vigorously develop ecological agriculture, reduce the use of chemical fertilizers and pesticides, and promote green agricultural and pastoral production. Rely on natural and cultural resources to design ecotourism routes and promote low‐carbon tourism development. Optimize population distribution, implement population gradient transfer in ecologically fragile areas, and guide concentration toward towns. Strengthen water and energy management, strictly control groundwater extraction, promote clean energy sources such as solar and wind power, and reduce regional ecological pressure.

## Author Contributions

**Zhikun Zhao:** conceptualization (equal), data curation (equal), formal analysis (equal), methodology (equal), visualization (equal), writing – original draft (equal). **Zhigang Fang:** formal analysis (equal), validation (equal), writing – review and editing (equal). **Yunlong Zhang:** methodology (equal), supervision (equal), validation (equal). **Chao Ma:** conceptualization (equal), funding acquisition (lead), methodology (equal), supervision (equal), visualization (equal), writing – review and editing (equal).

## Funding

This work was supported by the National Natural Science Foundation of China (No. U21A20108) and the Postdoctoral Fellowship Program of China Postdoctoral Science Foundation (No. GZC20240427).

## Conflicts of Interest

The authors declare no conflicts of interest.

## Supporting information


**Data S1:** Supporting information.

**Data S2:** Supporting information.

## Data Availability

All the required data are uploaded as Data S1 and S2.
