# Supplementary material for: Spatiotemporal Variation of Ecological Quality in the Yinshan Mountains Detected by MODIS Remote Sensing Indicators
Source: Ecol Evol. 2026 Jan 14;16(1):e72846. doi: 10.1002/ece3.72846 (PMC12802412; doi:10.1002/ece3.72846)
Supplement: Supplementary file 1 — Data S1: Supporting information. [file ECE3-16-e72846-s002.docx]

**Appendix**


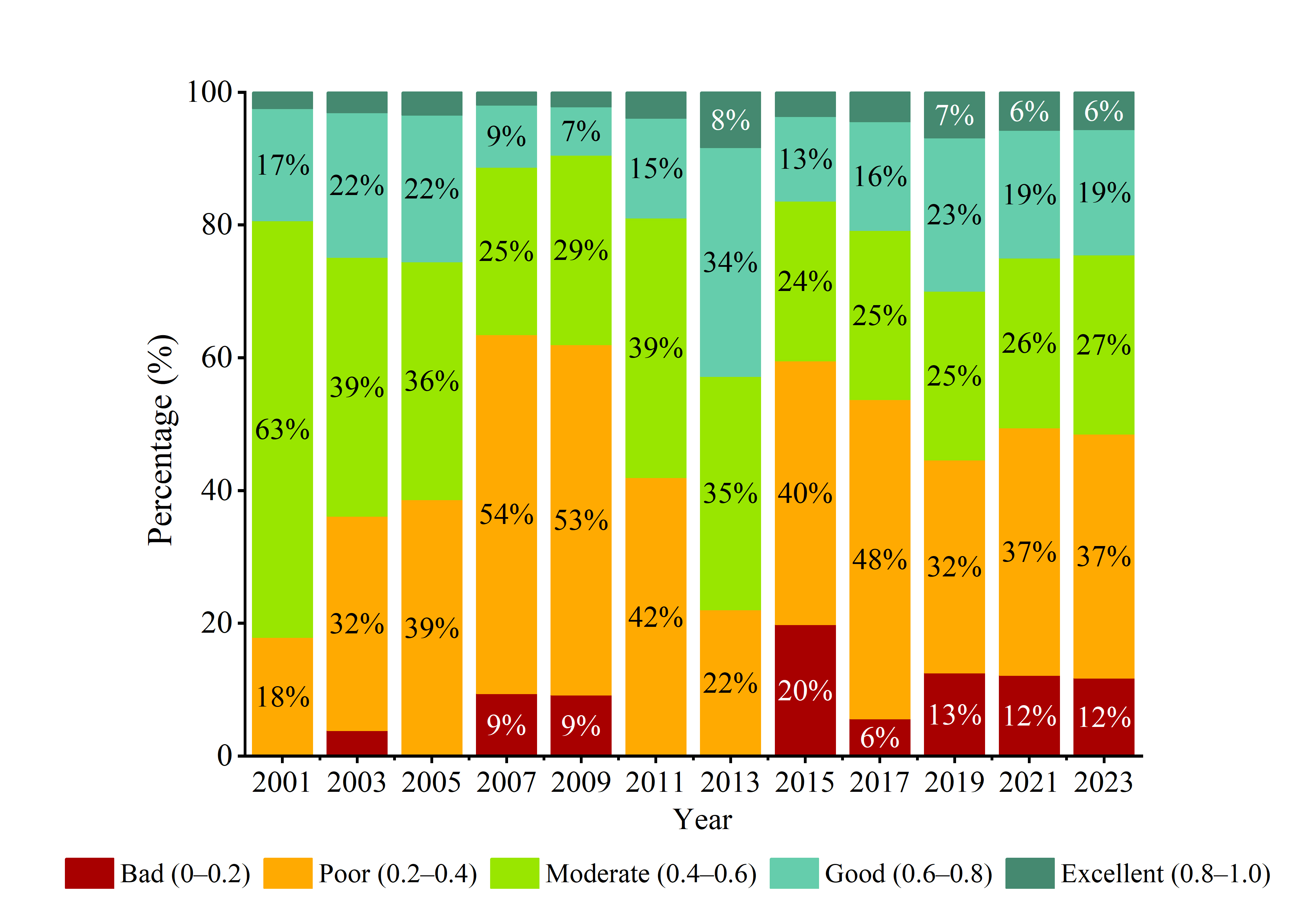


**Fig. A1.** Percentage of MODIS RSEI levels in the Yinshan Mountains from 2001 to 2023


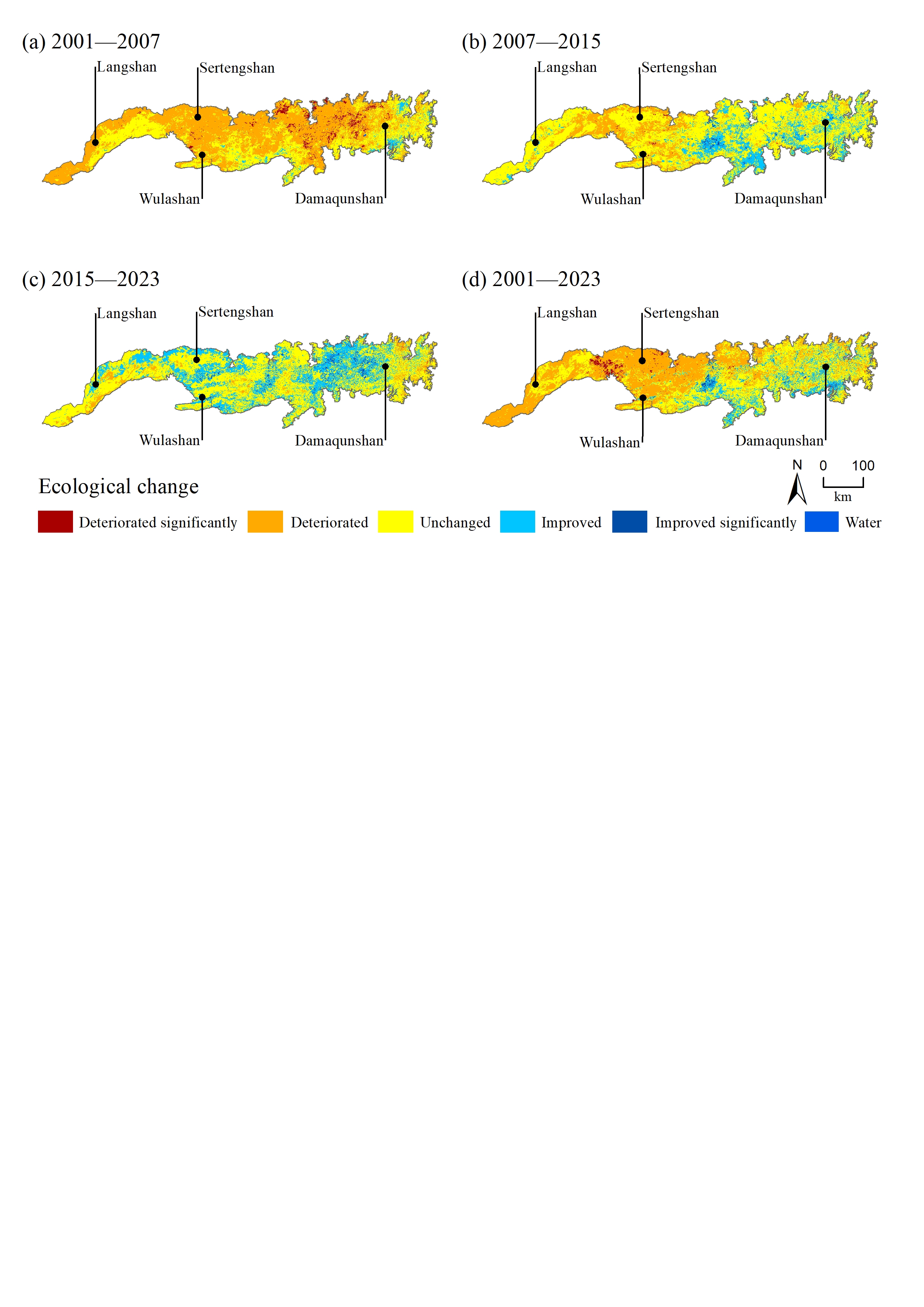


**Fig. A2.** Distribution of MODIS RSEI stage level changes in the Yinshan Mountains from 2001 to 2023


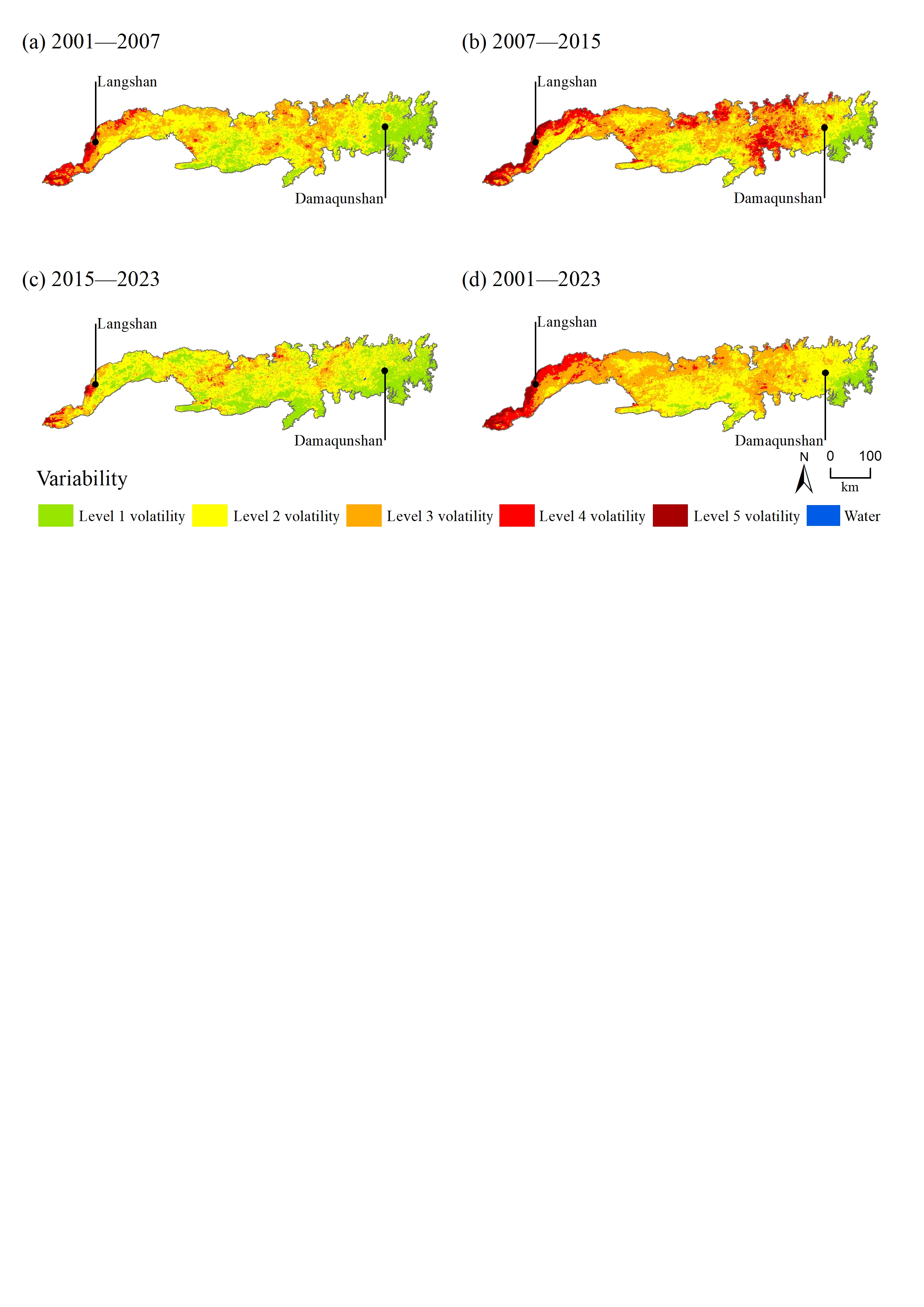


**Fig. A3.** Stability distribution of MODIS RSEI changes in the Yinshan Mountains from 2001 to 2023
