# Supplementary material for: Spatiotemporal Variation of Ecological Quality in the Yinshan Mountains Detected by MODIS Remote Sensing Indicators
Source: Ecol Evol. 2026 Jan 14;16(1):e72846. doi: 10.1002/ece3.72846 (PMC12802412; doi:10.1002/ece3.72846)

**Supplementary Materials**

**Fig. S1:** Spatial distribution of RSEI levels in the Yinshan mountains from 2001 to 2023


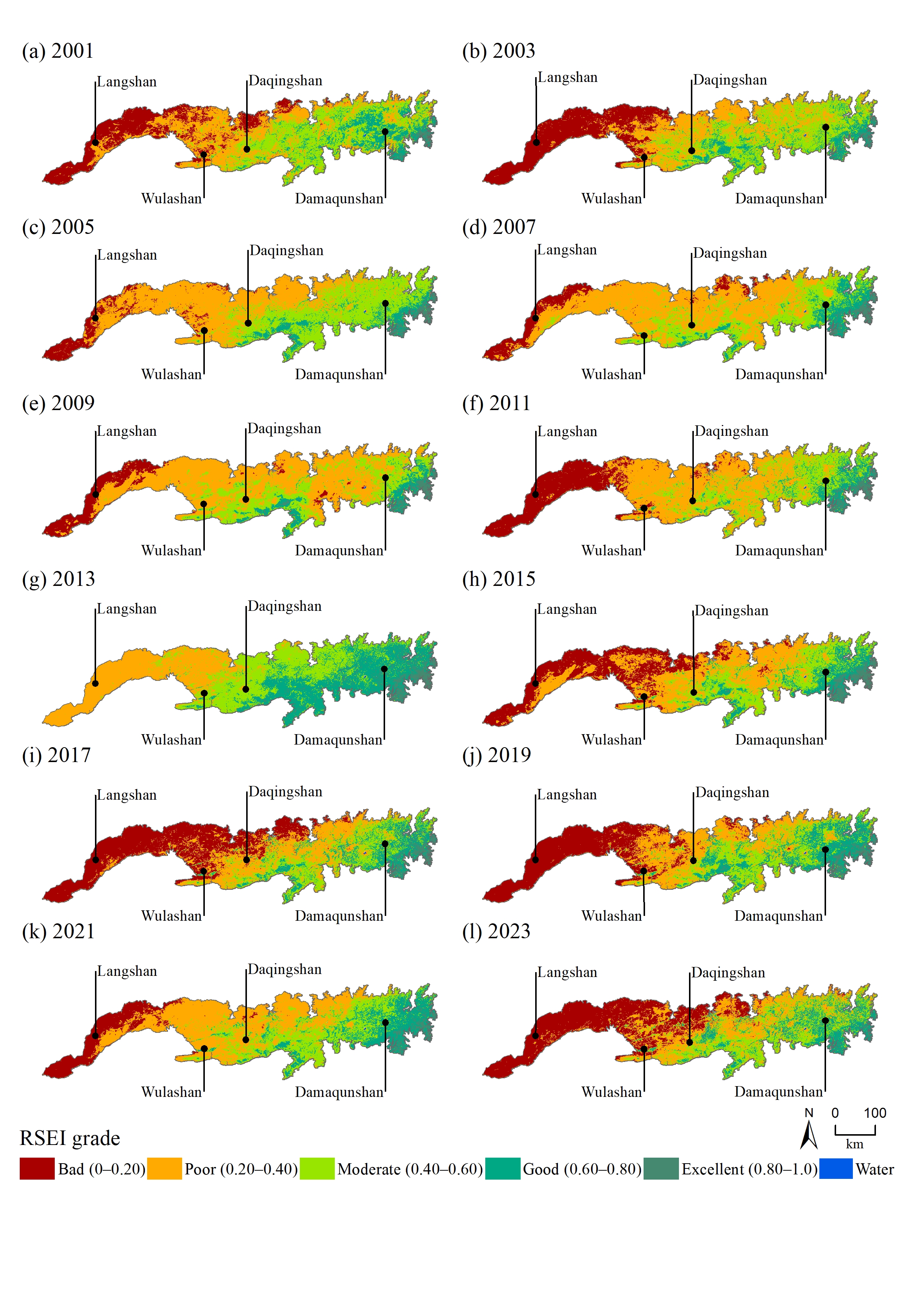


**Fig. S2:** Spatial differences between MODIS RSEI and RSEI in the Yinshan mountains from 2001 to 2023


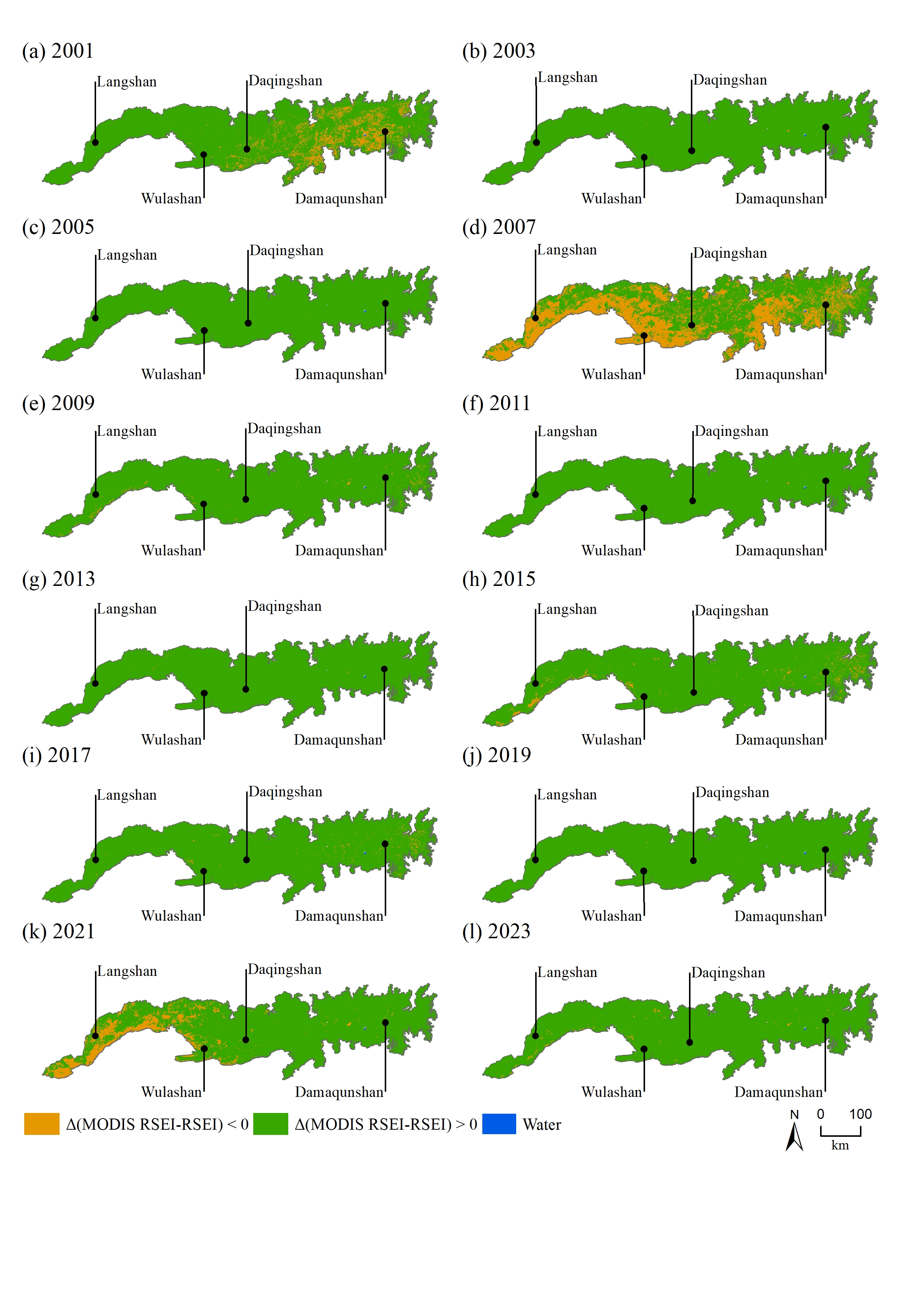

Supplement: Supplementary file 2 — Data S2: Supporting information. [file ECE3-16-e72846-s001.docx]
